# The Establishment of a Terrestrial Macroalga Canopy Impacts Microbial Soil Communities in Antarctica

**DOI:** 10.1007/s00248-025-02501-8

**Published:** 2025-02-13

**Authors:** Rodrigo Márquez-Sanz, Isaac Garrido-Benavent, Jorge Durán, Asunción de los Ríos

**Affiliations:** 1 Department of Biology and Geology, Physics, and Inorganic Chemistry, Rey Juan Carlos University, C/ Tulipán S/N, Móstoles, 28933 Madrid, Spain; 2 Department of Biogeochemistry and Microbial Ecology, National Museum of Natural Sciences (MNCN), CSIC, 28006 Madrid, Spain; 3 Departament de Botànica I Geologia, Fac. CC. Biològiques, Universitat de València, C/ Doctor Moliner 50, 46100 Burjassot, Valencia Spain; 4 Misión Biológica de Galicia (MBG), CSIC, 36143 Pontevedra, Spain

**Keywords:** Cryptogamic covers, Metabarcoding, Community ecology, Polar, *Prasiola*, Trebouxiophyceae

## Abstract

*Prasiola* is a genus of foliose green algae that forms extensive cryptogamic canopies that contribute to the greening of ice-free areas in the Antarctic tundra. To better understand the impact of *Prasiola* canopy establishment on colonization in these areas, this study compared the taxonomic and functional structures of bacterial and fungal communities in adjacent soils with and without extensive *Prasiola* colonization. DNA metabarcoding was employed to analyze the microbial community structure in these soils and in the canopy. Additionally, a phylogenetic study of *Prasiola* samples was conducted to characterize the taxonomic composition of the analyzed canopies, revealing the presence of *Prasiola crispa* (Lightfoot) Kützing and *P. antarctica* Kützing. Key soil attributes were assessed to examine the canopy’s influence. Higher pH and carbon, nitrogen, and organic matter contents were found in *Prasiola*-covered soils than in bare soils. Furthermore, *Prasiola* canopy establishment not only influenced abiotic soil properties but also shaped soil microbial community structure and its functions. For instance, while Actinobacteriota predominated in bacterial communities both within the *Prasiola* canopy and beneath it, Bacteroidota dominated in the bare soil. Despite significant variability across soil types, fungal communities showed a trend of higher abundances in certain Ascomycetes, such as Helotiales, Hypocreales, or Xylariales, in soils beneath *Prasiola* compared to bare soils. Regarding functional diversity, covered soils exhibited a statistically significant lower potential for bacterial methanogenesis and autotrophic CO_2_ fixation compared to bare soils. Finally, lichenized fungi, plant pathogens, and fungal wood saprotrophs tended to be more abundant in covered soils.

**Supplementary Information:**

The online version contains supplementary material available at 10.1007/s00248-025-02501-8.

## Introduction

Antarctica is the coldest and driest continent on Earth. Only about 0.37% of the continent is ice-free [1], mainly in maritime-influenced areas where a dense tundra covers the soil surface [2, 3], harbouring more than half of the total green vegetation of the continent [4]. Biological diversity and ecosystem functions in Antarctica are known to be less rich compared to other continents [5, 6]. From the vascular plant perspective, only two angiosperm species, *Deschampsia antarctica* Desv. and *Colobanthus quitensis* (Kunth) Bartl., are found in Maritime Antarctica [7–9]. The main primary producers in Antarctic terrestrial ecosystems are therefore cryptogams, a group that includes bryophytes (i.e., mosses, liverworts, and hornworts) and algae including lichenized lineages [10]. Due to their poikilohydric nature, lichens and mosses are more tolerant to extreme conditions, leading to significantly greater relative diversity and biomass in Antarctica compared to other ecosystems [3, 7, 8, 11–13]. On the other hand, the diversity of microorganisms in this continent appears to be much higher than previously estimated [14–18], although many Antarctic terrestrial areas have been seldom investigated. Thus, exploring the diversity of Antarctic microbial communities is essential for gaining insight into the continent’s total biodiversity and the factors that drive their spatial distribution.

The formation of biological soil crusts (biocrusts) on the soil surfaces in arid regions has been extensively studied [19]. These structures are formed by the association of various organisms, primarily mosses, lichens, and cyanobacteria [7, 20], which colonize soils in areas where water stress limits the development of vascular plants, such as deserts or polar areas [21]. It is estimated that 12% of the Earth’s surface is covered by biocrusts [22, 23], which contribute to soil development, atmospheric N_2_, and CO_2_ fixation and facilitate ecological succession by promoting the colonization by other organisms [19, 24, 25]. Recent studies have also demonstrated that the establishment of biocrusts modifies the diversity and composition of soil microbial communities beneath them [21, 22, 26, 27]. As mentioned above, dense cryptogamic canopies are also common in Antarctica, where they develop on soils in a less compact manner compared to desert biocrusts, thus failing to form a compact structure that aggregates with the soil. However, structural and functional similarities to biocrusts can still be observed as Antarctic cryptogamic communities are primarily composed of mosses, liverworts, and/or lichens [19, 27]. Despite this, little is known about the diversity and structure of microbial communities and soil attributes when macroalgae dominate these cryptogamic covers.

Among terrestrial macroalgae, several species of the genus *Prasiola* (C. Agardh) Meneghini (Trebouxiophyceae, Chlorophyta) are commonly found in Antarctica [28]. With approximately 35 species described, *Prasiola* mostly comprises macroscopic green algae that form visible leafy thalli [28, 29]. In Antarctic terrestrial ecosystems, these macroalgae contribute significantly to primary production [30] and have been shown to exert a significant impact on soil biogeochemical attributes [26]. Antarctic *Prasiola* species frequently occur in ice-free areas with maritime influence, where soils are frequently enriched with nitrogen and organic matter from bird colonies [29]. These macroalgae often form extensive canopies, due to their adaptation to extreme conditions, such as freezing, UV radiation, water stress, and salinity [31, 32]. Further, studies show that rising temperatures in Maritime Antarctica [33] and the increase in ice-free soils due to accelerated glacier retreat may lead to the expansion of these macroalgae [34]. However, the effects of these communities’ establishment on soil ecosystems are largely unknown. Thus, assessing the effects of *Prasiola* canopy establishment on microbial community diversity and composition, as well as their influence on soil attributes, is essential for predicting how these terrestrial ecosystems will respond to warming in polar regions.

The main goal of the current work was to analyze the influence of *Prasiola* canopies on the taxonomic structure and function of Antarctic soil microbial communities, as well as on abiotic soil attributes. For this purpose, a study area with extensive colonization by terrestrial *Prasiola* spp. was selected and DNA metabarcoding was employed to explore bacterial and fungal communities. The findings of this study will offer insights into the functioning of this unique Antarctic terrestrial ecosystem, which is likely to be affected by future scenarios of climate change [23, 28].

## Material and Methods

### Site Description

**Fig. 1:**
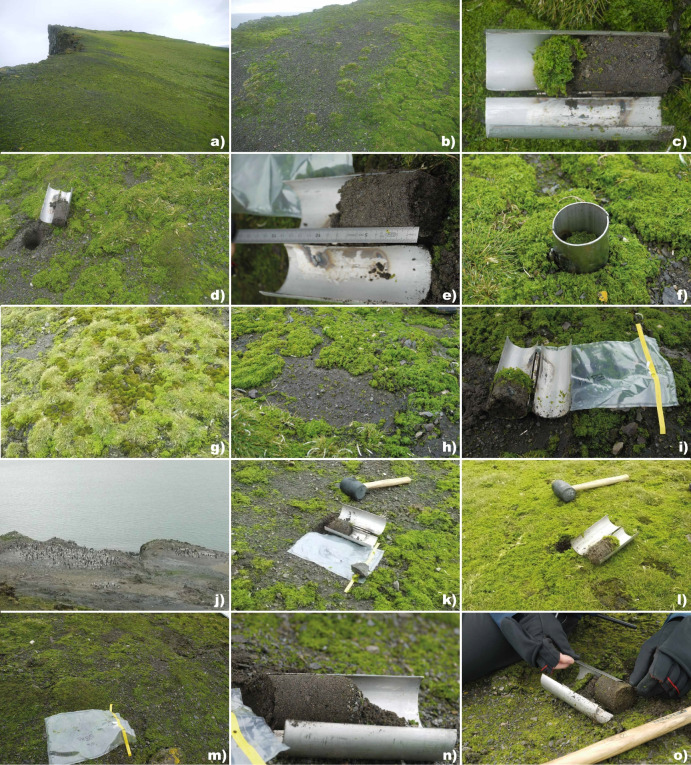
**a** Sampling area showing soils covered by a canopy dominated by macroalgae *Prasiola* spp. **b** Sampling point 1. **c** *Prasiola*-covered soil core collected at sampling point 1 (APH1). **d** Sampling point 2. **e** Bare soil core collected at sampling point 2 (ABH2). **f** *Prasiola*-covered soil core within the cylinder at sampling point 2 (APH2). **g** *Prasiola* canopy showing presece of bryophytes and *Deschampsia antarctica*. **h** Sampling point 3. **i** Bare soil core collected at sampling point 3 (ABH3). **j** An area near the study site, where a penguin colony is present. **k** Bare soil core collected at sampling point 4 (ABH4). **l** *Prasiola*-covered soil core collected at sampling point 4 (APH4). **n** Bare soil core collected at sampling point 5 (ABH5). **o** Depth test performed at sampling point 5 (APH5)

Samples were collected from five sampling points within an area of approximately 1885 m^2^, extensively colonized by *Prasiola* in Hannah Point Bay (Livingston Island, South Shetland Islands; Table 1; Fig. 1). This area is characterized by its closeness to the sea and the presence of nearby petrel nests, which may contribute to the accumulation of organic matter on the soils. As a coastal tundra ice-free zone, the soils are covered by a patchy but extensive canopy comprising various bryophytes, the terrestrial algae *Prasiola* spp., and scattered tufts of *Deschampsia antarctica*. Climatic data were not collected directly; however, based on records from the Juan Carlos I Spanish Antarctic AEMET meteorological station, located approximately 10 km from the sampling area on the same island, the average temperature is estimated to be around − 1.5 °C, with annual precipitation of 400 mm.

**Table 1:** GPS coordinates and altitude for each sampling point

Sampling point	Sample labels	Longitude (S)	Latitude (W)	Altitude (metres)
1	AHB1/AHP1	62° 39′ 15.32″	60° 36′ 27.08″	378
2	AHB2/AHP2	62° 39′ 14.96″	60° 36′ 27.83″	372
3	AHB3/AHP3	62° 39′ 15.94″	60° 36′ 32.03″	381
4	AHB4/AHP4	62° 39′ 15.37″	60° 36′ 27.44″	377
5	AHB5/AHP5	62° 39′ 15.23″	60° 36′ 28.05″	375

### Sample Collection and Storage

Two composite soil samples, each from a 0–5 cm depth, were collected at each of the five sampling points: one from soil beneath the *Prasiola* canopy and one from bare soil (without canopy). A minimum distance of 10 m (and up to 83 m) was maintained between sampling points to minimize interference from factors other than the macroalgal canopy on microbial communities. Photographs were taken at each sampling point to document and characterize the canopy.

Each composite sample was formed by collecting three soil cores using a 5-cm diameter cylinder. The soil cores were placed in sterile Whirl–Pak^®^ bags directly from the cylinder using sterile gloves. To prevent cross-contamination, the cylinders were disinfected with 96° ethanol between samples. The algal canopy was removed using a sterile blade before placing *Prasiola*-covered samples in the bags and was stored separately. Soils were immediately sieved through a 2-mm mesh and thoroughly homogenized under sterile conditions in a laminar flow hood at the laboratories of the Spanish Antarctic Base Juan Carlos I. Then, 1 g of each type of the sieved soils and of the *Prasiola* canopies was added immediately to tubes containing RNAlater (Sigma, Life Science). These tubes were preserved at 5 °C until DNA extraction began. The remaining soil, which was used for the characterization of soil abiotic attributes, was air-dried at room temperature and stored at 5 °C until further processing.

### Analysis of Soil Attributes

Soil samples were grinded in a mortar prior to measuring pH, organic matter, nitrogen (N), and carbon (C) contents. Soil pH was measured using a pH-meter (Crison MicropH 2001) at a soil/water ratio of 1/2.5 (mass/volume). Organic matter content was estimated by weight difference before and after ignition at 450 °C according to Nelson and Sommers [35] and expressed as a percentage. Finally, C and N contents in 100 g were measured by dry combustion using a macro combustion analyzer (LECO TruSpec CN).

### Taxonomy and Phylogeny of Canopy *Prasiola* Species

Taxonomic identification of *Prasiola* species was achieved using molecular phylogenetics, as the use of morphological characters to diagnose species in this genus is often problematic [30, 36]. The gene marker *tuf*A (elongation factor Tu) was amplified because it was proposed as a green algal barcode by Saunders and Kucera [37] and proven to be suitable for delimiting *Prasiola* species [29, 36, 38].

Total genomic DNA was extracted from 2 × 2 mm sections of hydrated thalli collected in each *Prasiola* cover. These fragments were washed and examined under a stereomicroscope to remove potentially contaminating microalgae. Then, they were manually grinded, and DNA was extracted using the Speedtools Tissue DNA extraction kit (Biotools^®^ B&M Labs., S.A) following the manufacturer’s recommendations. Subsequently, a nested PCR amplification of the *tuf*A region was performed using the Illustra Ready-To-Go GenomiPhi V3 DNA amplification kit (GE Healthcare Bio-Sciences, Pittsburgh, Pennsylvania, USA). In the first and second PCR rounds, universal primers tufA-F and tufA-R [39] and *Prasiola*-specific forward and reverse internal primers [36] were used and PCR products were purified using QIAGEN spin columns (Qiagen^®^). Finally, complementary DNA strands were sequenced in MACROGEN (Madrid) and electropherograms were edited using SeqmanII v.5.07^©^ (Dnastar Inc.).

The *tuf*A dataset for phylogenetic analyses consisted of six sequences obtained from the five sampling points, in addition to 91 sequences downloaded from GenBank (accessible via https://www.ncbi.nlm.nih.gov/genbank/; Table S1). These included sequences of *Rosenvingiella* P.C. Silva species, which were selected as outgroups to root the phylogenetic tree following Garrido-Benavent et al. [36]. Sequence alignment was carried out in AliView software [40], using the MUSCLE default algorithm [41]. Since the *tuf*A marker includes a coding region, the alignment was divided into three partitions to run the analyses so that each codon position was taken into consideration. With the initial 97 sequences, a maximum likelihood (ML) analysis was performed using IQ-TREE [42, 43], keeping the default parameters, where it automatically selects the nucleotide substitution model that best fits each alignment partition. A clade was considered statistically supported when its ultraBootstrap value [44] was equal to or greater than 95%. For Bayesian inference (BI), nucleotide substitution models that best fitted alignment partitions were evaluated in PartitionFinder 2.1.1 [45] using the corrected Akaike Information Criterion (AICc). Bayesian analysis was then performed in MrBayes v. 3.2.7 [46] and consisted of two runs of eight MCMC chains each that were carried out over 20 million generations, starting with a random tree and sampling one tree every 100 generations. A burn-in proportion of the first 50% trees was discarded when calculating the consensus tree. The chains were assumed to converge properly when the standard deviation of the group frequencies fell below 0.01. The consensus tree (50% majority rule) and the posterior probability (PP) value of the nodes were calculated and considered statistically supported when PP was equal to or greater than 0.95. Subsequently, after checking the congruence of the clades revealed by ML and BI inferences, both Bayesian posterior probability and ultraBootstrap values were plotted on the ML tree.

### DNA Extraction from Soil Samples and Sequencing

Approximately 0.35 g of both soil samples and *Prasiola* canopy preserved in RNAlater was retrieved for DNA extraction using the DNeasy PowerSoil extraction kit (MO BIO Laboratories, Carlsbad, USA) following the recommended standard protocol. DNA concentration and purity were checked using a NanoDrop ND 1000 spectrophotometer (Thermo Fisher Scientific TM). Then, DNA amplification was done using the barcoding universal primers 515F-806R [47] spanning the V4 hypervariable region of the bacterial 16S rRNA [48] and the primer pair ITS1F_KYO2-ITS2_KYO2 [49] spanning the *ITS1* region of the fungal nuclear ribosomal Internal Transcribed Spacer [50]. Both PCR protocols followed the Earth Microbiome Project (http://www.earthmicrobiome.org-standards/). In the case of the macroalgal canopy, only the bacterial community was studied by metabarcoding. PCR products were sent to the Arizona State University Genomics Service (ASU Genomics Core) for library preparation and paired-end sequencing on an Illumina MiSeq sequencer (version 2 module, 2 × 250 bp), following the manufacturer’s instructions. Raw reads were demultiplexed and barcode sequences were removed by the sequencing centre. The data sets generated for this study were deposited in the NCBI Sequence Archive with BioProject number PRJNA1212754.

### Bioinformatic Analyses for Bacterial and Fungal Community Profiling

Illumina sequence data for bacteria were processed with QIIME2 v. 2021.11 [51], using a modified version of the Divisive Amplicon Denoizing Algorithm pipeline (DADA2) [52]. Briefly, compressed *gz* files were transformed into FastQC [53] and MultiQC [54] files, considering eligible reads from both direct and reverse primers to check for read quality (Fig. S1). Sequence ends were trimmed, selecting between positions 10 and 250 from direct reads and 10 and 240 from reverse reads. Then, denoizing was performed, and a *biom* table was obtained containing ASV abundances per sample. The corresponding metadata, which consisted of information associated with each sample, was added to the abundance table. Subsequently, another filtering step was applied to the *biom* table to remove ASVs that were present in less than two samples. This step reduced the initially inferred 4249 ASVs to 1817, although the total number of reads varied little (from 1425153 to 1343205 reads). Taxonomic assignment was then performed using the SILVA database (https://www.arb-silva.de/) [55]. Finally, the resulting *biom* table was filtered to remove chloroplast, archaea, or mitochondria sequences.

Fungal *gz* data files were transformed into FastQC and then into MultiQC files in the QIIME2 virtual environment to check for read quality. Due to low-quality scores of reads obtained with the reverse primer, only forward reads were used in the analysis (Fig. S1). The loss of information due to filtering reads from one of the two primers does not significantly affect subsequent analyses derived from the use of these data [56]. Subsequent analyses were performed using DADA2 within the Microbiome Helper amplicon v0.3 environment [57]. Additionally, the ITSx program [58] was run in a loop to extract exact ITS sequences from the reads obtained by Illumina sequencing (further details and script in Garrido-Benavent et al. [59]). Sequences with less than 50 bp were removed. Taxonomic assignment was performed using the UNITE database [60]. The resulting ASV abundance table was converted into *biom* format, adding metadata with information from each sample.

For both bacteria and fungi, the R-based [61] online tool Microbiome Analyst [62] was used to analyze the *biom* tables. Taxonomic profiles were represented by relative abundance histograms highlighting the most abundant groups. The remaining groups, which showed a lower relative abundance, were grouped into a single category named “Others”. The bacterial phylum *Proteobacteria* was subdivided into classes *Alpha*- and *Gammaproteobacteria*. Sample AHB3 was not included in the analyses because it had a very low number of sequence reads. Venn diagrams were generated using the online tool *jvenn* [63] to show shared and unique ASVs to each of the different sample types.

### Statistical Analyses

For key soil attributes Shapiro–Wilk and Levene’s tests were employed to test for normality and equality of variances, respectively, and ANOVA or Kruskal–Wallis tests to assess differences in soil attributes among soil types employing R functions *aov* and *Kruskal.test*, respectively. Statistically significant differences for relative abundances of bacteria and fungi and fungal ecological functions were tested using the non-parametric Kruskal–Wallis test and Mann–Whitney tests, using the R functions *Kruskal.test* and *wilcox.test*. To test for statistical differences in the abundance of ecological functionalities, Welch’s test of R package *Aldex2* [64] was used to analyze the results of Picrust2 in bacteria. Differences were considered significant when *p*-values were less than 0.05.

### Microbial Diversity

Richness was measured by calculating the number of ASVs present in each sample. The *α*-diversity indexes Shannon’s (H′) and Simpson’s (D) were calculated based on rarefied sample tables using Microbiome Analyst [62]. The evenness was expressed by Pielou’s Evenness index [65] with the R *phyloseq* script [66]. *β*-diversity was calculated by the Bray–Curtis index [67] using a *biom* table normalized by the Cumulative Sum Scaling method (CSS) [68]. A principal coordinate analysis (PCoA) [69] and a non-metric multidimensional scaling (NMDS) [70] were performed on the basis of Bray–Curtis dissimilarities to illustrate differences in community composition across sample types. To assess statistical support for any observed differences, a permutational multivariate analysis of variance (PERMANOVA) [71] and an analysis of similarities (ANOSIM) [72] were performed. Finally, the relationships between soil attributes and bacterial and fungal community structure were examined and graphically represented using a distance-based redundancy analysis (db-RDA) [73] with the R *vegan* package [74]. Before building the constrained ordination plots, log10 was used to transform attribute variable organic matter and C contents, and the C/N ratio.

### Functional Diversity

The Picrust2 algorithm [75] implemented in QIIME2 [51] was employed to predict the metabolic potential of the bacterial communities. As output, a functional profile consisting of 405 metabolic pathways was obtained and then classified into 48 categories based on the MetaCyc database [76]. The threshold for metabolic pathway significance was set to 0.05. To visualize results, boxplots of 5 selected functional categories were constructed with R function *boxplot.* Principal component analysis (PCA) was performed considering all metabolic pathways retrieved using the STAMP software [77], as recommended by Picrust2 developers.

The database Fungal Traits [78] was used to assign a potential role to ASVs with genus-level affiliation. This process produced a table of ecological functions based on genus-level ASV abundance, including a group with undetermined ecosystem functions.

## Results

### Taxonomic Characterization of Canopy-Forming *Prasiola* Species

**Fig. 2:**
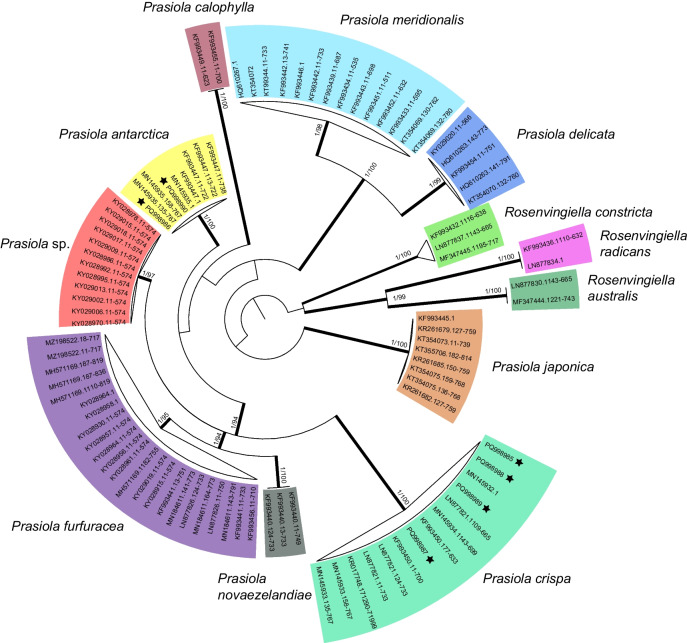
ML phylogenetic tree depicting evolutionary relationships among *Prasiola* species and clade membership of specimens forming the Antarctic canopies (indicated with a star symbol). Thickened branches show nodes supported by ultraBootstrap values (ML analysis) of Bayesian posterior probabilities (BI analysis)

Both maximum likelihood (ML) and Bayesian inference (BI) phylogenetic analyses using *tuf*A sequence data (Fig. 2) showed that the *Prasiola* canopy at Hannah Point comprised two species, *Prasiola crispa* (Lightfoot) Kützing (sampling points 1, 3, and 4) and *P. antarctica* Kützing (sampling point 2), while at sampling point 5 both species occurred.

### Soil Attributes

**Fig. 3:**
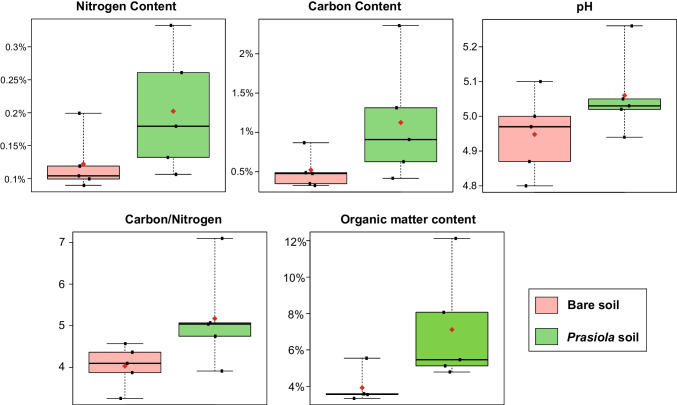
Boxplots showing average (represented by rhombi) and standard errors of soil attributes in bare soils and soils under a *Prasiola* canopy

Although no significant differences were observed (likely due to high variability), average values of C, N, and organic matter content, as well as pH and the C/N ratio, were consistently higher in soil samples collected under a *Prasiola* canopy compared to bare soils (Fig. 3), with significance values close to the 0.05 threshold in most cases (Table S2).

### Microbial Community Structure of Soils and *Prasiola* Canopy

**Fig. 4:**
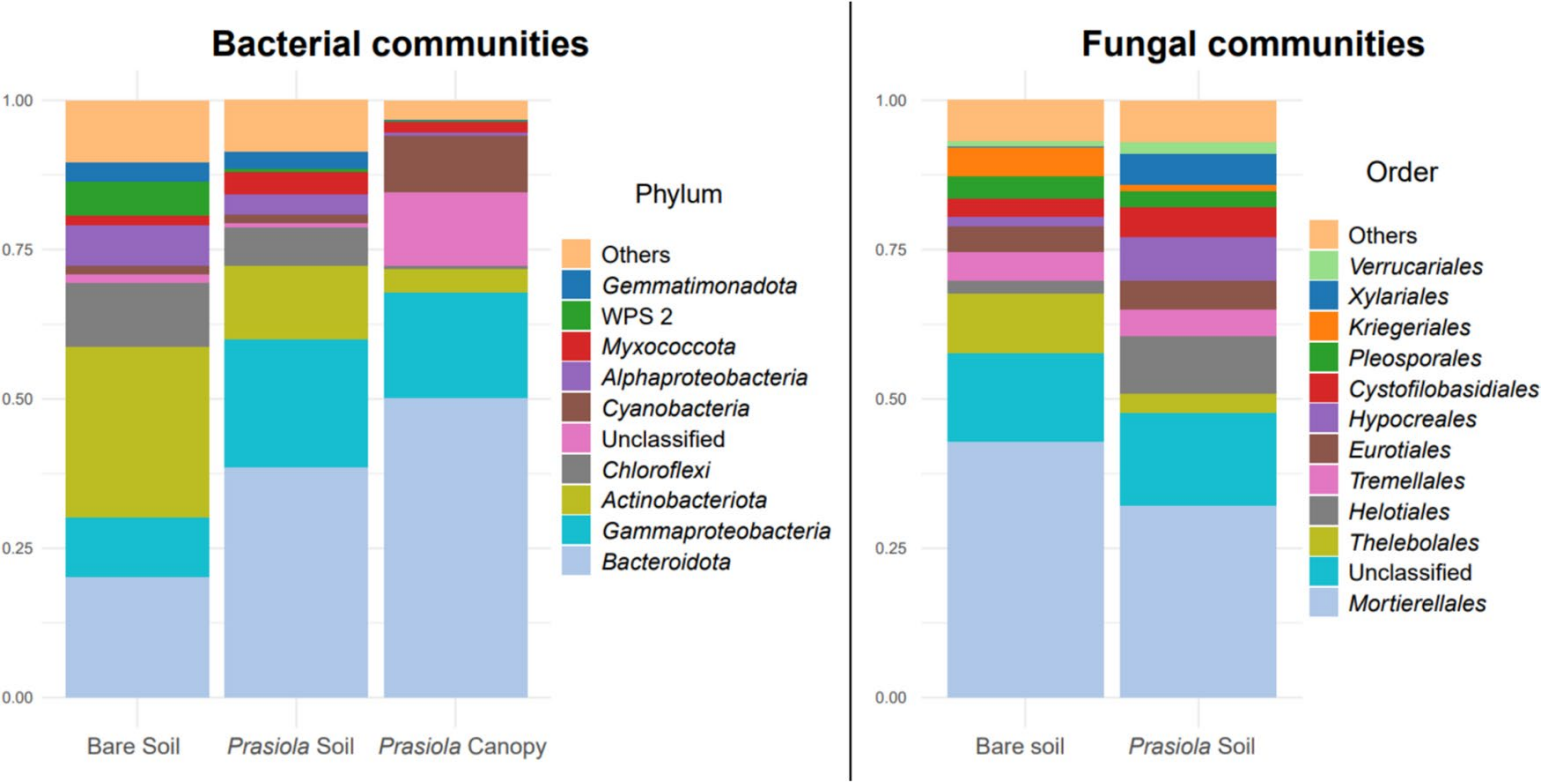
Relative abundance of bacterial phyla and fungal orders revealed by high-throughput Illumina sequencing. The category “Others” includes groups representing less than 0.25% of the total relative abundance in bacteria and less than 0.005% in fungi

After filtering sequences, the resulting data set consisted of 1792 bacterial ASVs based on 1,210,688 reads and 174 fungal ASVs based on 228,954 reads. The bacterial phylum Bacteroidota prevailed in both the *Prasiola* canopies and the soils beneath them*,* followed by Gammaproteobacteria (Fig. 4, Fig. S2). In contrast, the predominant phylum in bare soil communities was Actinobacteriota, followed by Bacteroidota.

Statistically significant differences were found between bare soils and *Prasiola*-covered soils in the relative abundances of the phyla Actinobacteriota and WPS 2 (Table S3), both of which were more abundant in bare soil communities. Conversely, Bacteroidota, Myxococcota, and the class Gammaproteobacteria showed higher abundances in soils under *Prasiola.* We observed significantly higher relative abundances of Alphaproteobacteria, Actinobacteria, Chloroflexi, Myxococcota, Gemmatimonadota, and Eremiobacteria in the covered soil, whereas unclassified ASVs were more abundant in the canopy. The average relative abundance of Cyanobacteria was higher in the *Prasiola* canopy than in both soil types, although significant differences were not found due to substantial variability among samples (Table S3, Fig. S2).

In general, the relative abundances of bacterial phyla were quite consistent among samples within the same category, with only slightly higher variability observed in the canopy samples. In contrast, fungal profiles exhibited significant variability across sample types (bare soil and soil under *Prasiola* canopy; Fig. S2). Although no statistically significant differences in relative abundances were found among the fungal orders (Table S4), certain ascomycetes such as Helotiales, Hypocreales, or Xylariales, showed higher average relative abundance in soils under *Prasiola* compared to bare soils*.* Conversely, Mortierellales (the most prevalent order in both edaphic fungal communities) and Kriegeriales tended to be more abundant in bare soils.

**Fig. 5:**
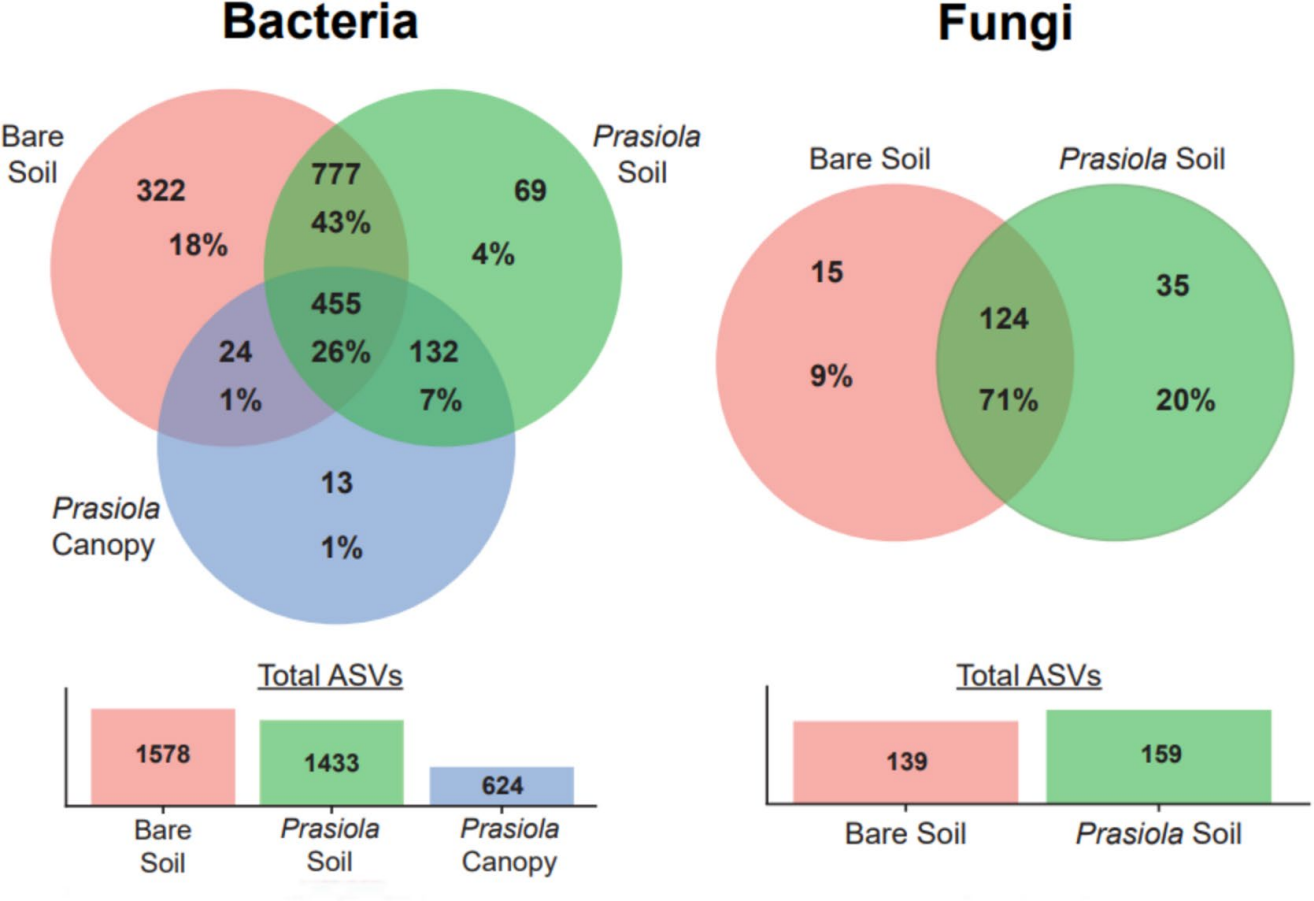
Venn diagrams representing the number and proportion of shared and unique ASVs for each sample type in bacterial and fungal communities

The analysis of bacterial ASVs shared among different sample types revealed that most of them were not specific to any sample type (1388 ASVs, 77%; Fig. 5). Out of the shared ASVs, the majority were found in both soil types (777 ASVs, 43%) or were ubiquitous across all samples (455 ASVs, 26%). The *Prasiola* canopy shared significantly more ASVs with the soil beneath (132 ASVs, 7%) than with the bare soil (24 ASVs, 1%). Bare soil exhibited the highest number of unique bacterial ASVs (322 ASVs, 18%), followed by *Prasiola*-covered soil (69 ASVs, 4%), while the macroalgal canopy contained the lowest percentage of unique ASVs (13 ASVs, 1%). Regarding fungal communities, both soil types shared a substantial proportion of ASVs (124 ASVs, 71%; Fig. 5), but *Prasiola*-covered soil contained more unique ASVs (35 ASVs, 20%) compared to the bare soil (15 ASVs, 9%).

### Microbial Diversity

**Fig. 6:**
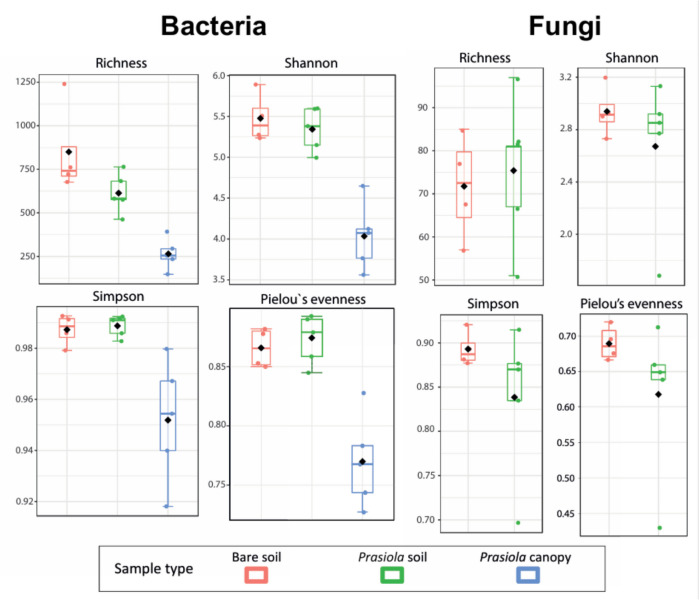
*α-*diversity indexes for bacterial and fungal communities in the different sample types. Mean values are represented by rhombi

Bacterial ASV richness was significantly higher in soils compared to the *Prasiola* canopy (Fig. 6, Table S5). The Shannon and Simpson diversity indexes, as well as Pielou’s evenness, showed similar values in both soil types. However, significantly lower values, accompanied by a greater variability between samples, were observed in the *Prasiola* canopy (Fig. 6). No significant differences were detected for fungal diversity (Table S6). Fungal ASV richness was slightly higher in *Prasiola-*covered compared to bare soil, whereas the Simpson and Shannon indexes and Pielou’s evenness showed the opposite trend. Fungal communities were highly variable in terms of richness, evenness, and Shannon and Simpson diversity indexes within each sample type, except for Simpson and Shannon indexes and Pielou’s evenness values from bare soil that were quite homogeneous (Fig. 6).

**Fig. 7:**
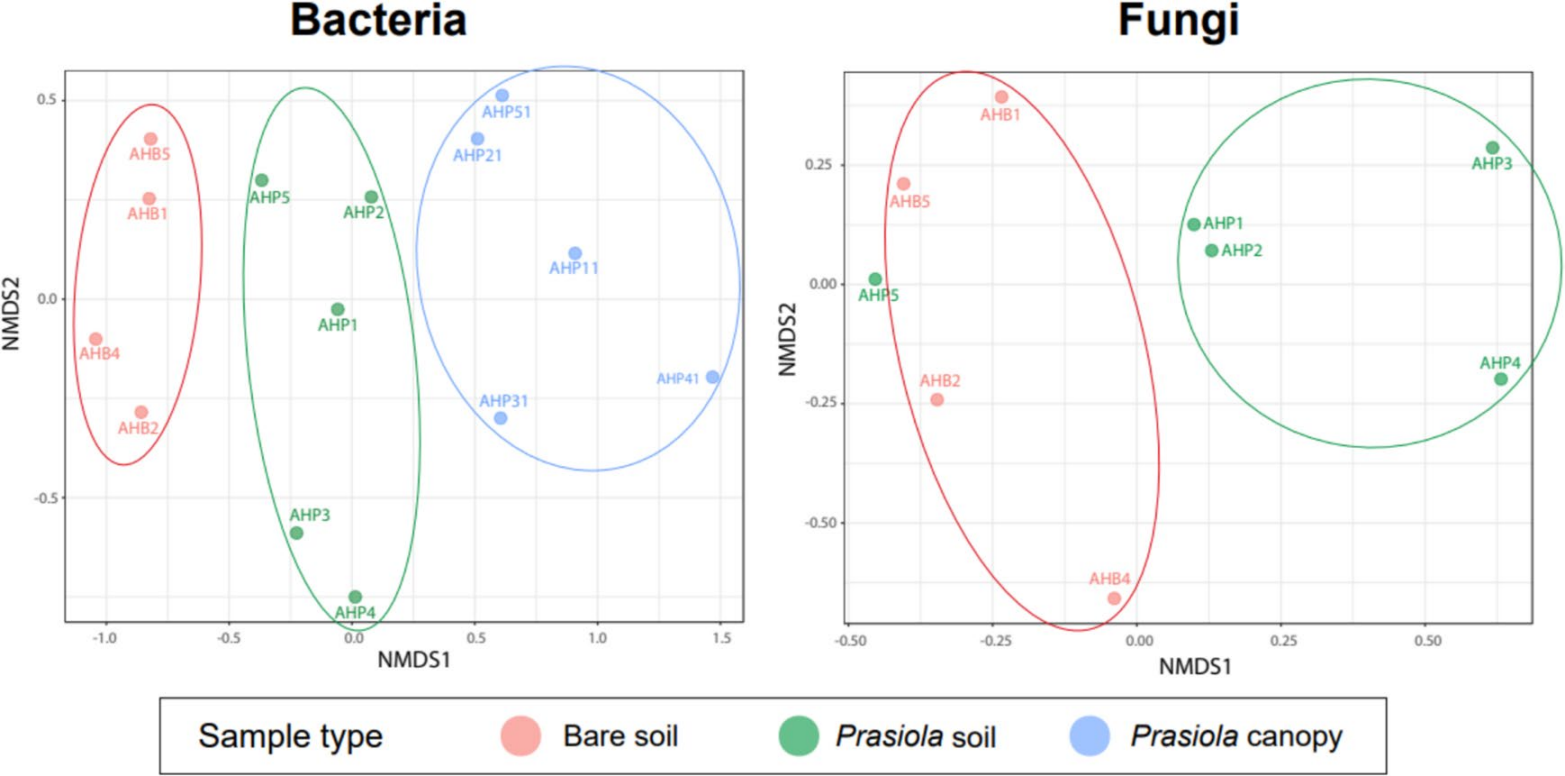
Non-metric multidimensional scaling (NMDS) ordination plots for bacterial and fungal communities in different sample types based on Bray–Curtis dissimilarities. Stress values were 0.06 and 0.07 for bacterial and fungal NMDSs, respectively

NMDS ordinations showed a distinct segregation of bacterial communities among the three sample types (Fig. 7), with the non-parametric ANOSIM test showing statistically significant differences between groups (*p* < 0.001). In contrast, the NMDS ordination of fungal communities revealed greater dispersion within sample types compared to bacterial ones, but also separated samples from bare and *Prasiola*-covered soils (Fig. 7). Only one sample (AHP5) of the latter category clustered with bare soil samples. However, the ANOSIM analysis did not find statistically significant differences between fungal communities from two sample types.

**Fig. 8:**
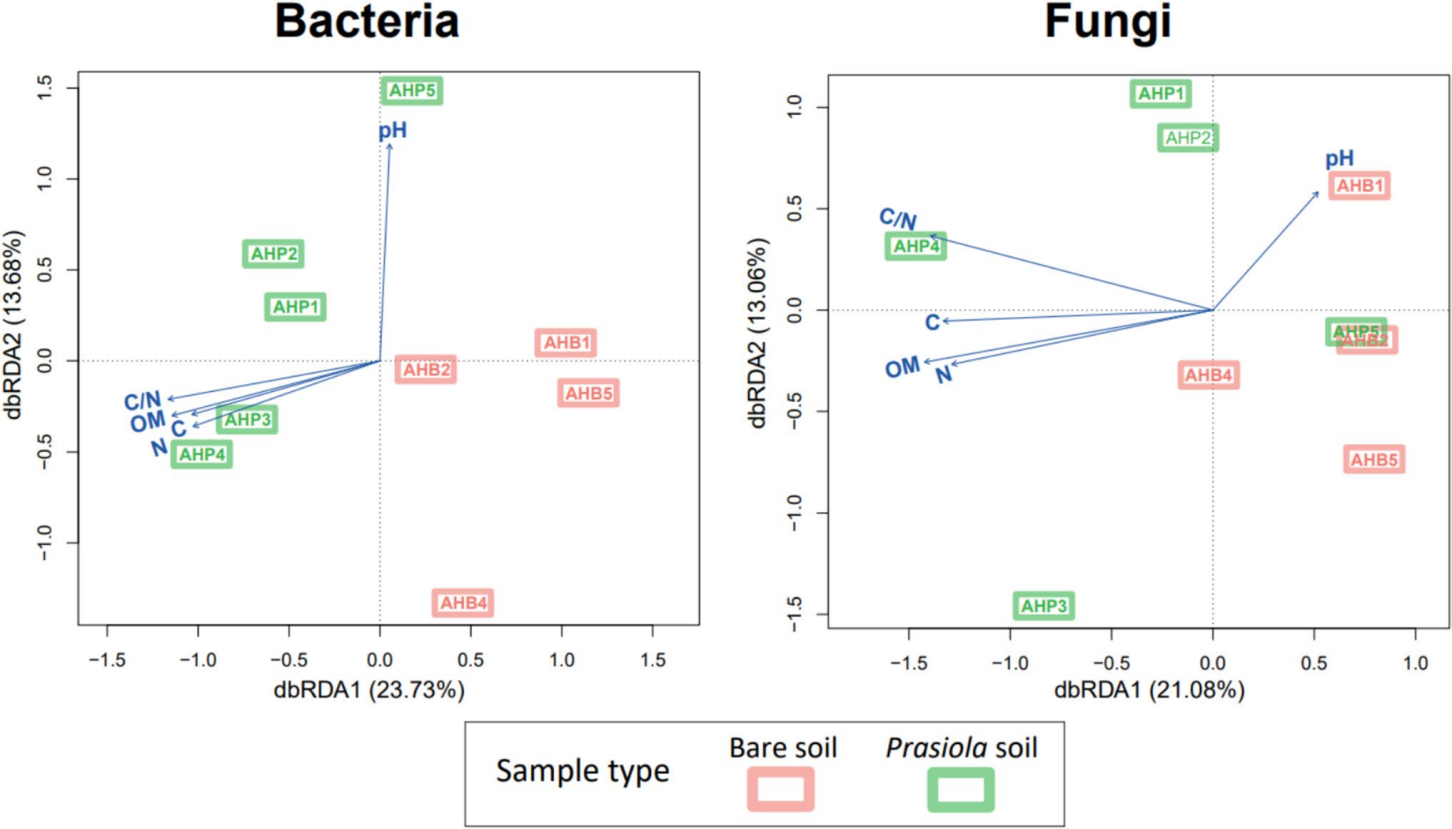
Distance-based redundancy analysis with selected edaphic variables that explained most of the variability between bacterial and fungal communities

The db-RDA pattern for bacterial and fungal communities highly resembles the ordination obtained in NMDS. Total variation explained by dbRDA1 and dbRDA2 axes was 37.41% and 34.14%, for bacterial and fungal communities and soil attributes (Fig. 8). Soil pH showed a positive correlation with db-RDA1 and db-RDA2 axis, while organic matter, N and C, and the C/N ratio exhibited negative correlations along the db-RDA1 axis. These factors effectively separated bacterial and fungal communities in bare soils from those developing beneath *Prasiola* canopies.

### Functional Microbial Community Structure of Soils

**Fig. 9:**
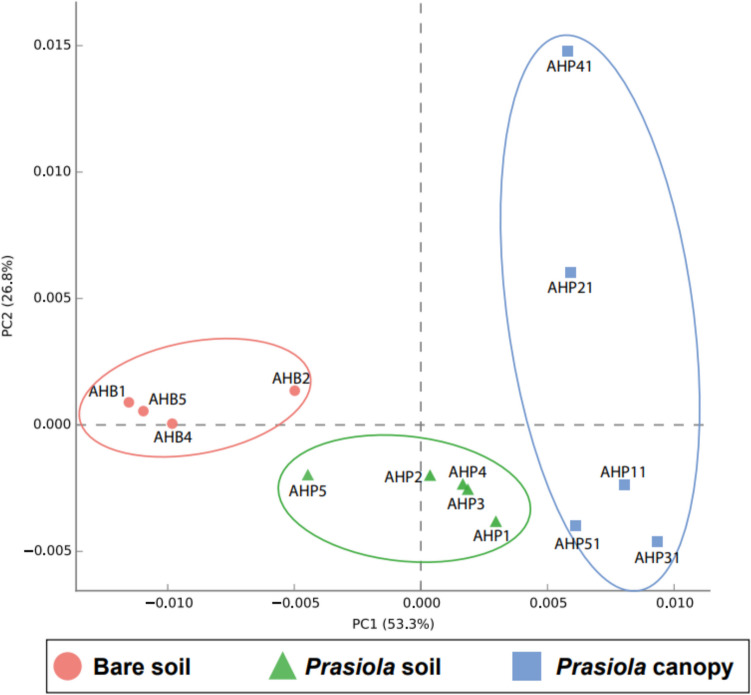
Principal component analysis generated using STAMP software for bacterial communities based on the abundances of 405 metabolic pathways resulting from Picrust2

The PCA ordination of potential metabolic pathways in bacterial ASVs clearly separated communities from the three sample types (Fig. 9) and revealed a greater variability in samples from the *Prasiola* canopy. Statistically significant differences (*p*-value < 0.05) were found in 104 out of 405 (~ 25%) pathways between the two soil types and in 223 out of 405 (~ 55%, Table S7) between the canopy and the soil beneath.

**Fig. 10:**
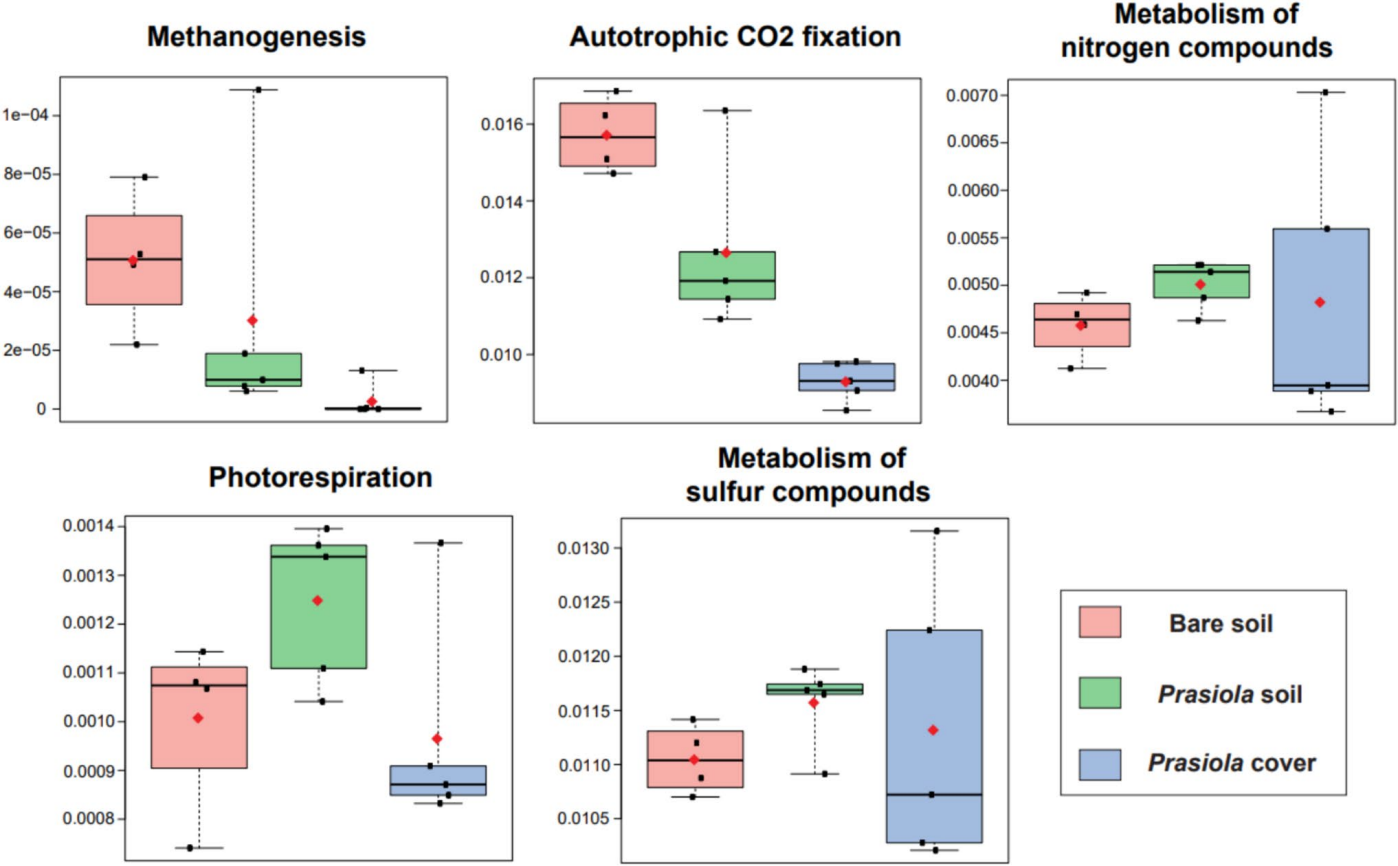
Relative abundance of selected functional categories generated by Picrust2 for bacterial communities from different sample types. Mean values are represented by rhombi

The 405 pathways were manually categorized into 48 functional groups based on the Metacyc pathways database [76]. Among these functional categories, specific pathways related to soil functioning were selected based on their correlation with measured abiotic attributes in this study: autotrophic CO_2_ fixation, metabolism of nitrogen compounds, metabolism of sulphur compounds, methanogenesis, and photorespiration. Statistically significant differences among sample types were found for methanogenesis and autotrophic CO_2_ fixation (Table S8), both of which exhibited higher potential in bare soils, particularly when compared to the *Prasiola* canopy, which showed minimal potential values, particularly for methanogenic potential (Fig. 10). It is also noteworthy that there was a trend toward higher potential for photorespiration in covered soils compared to bare soils and canopy.

**Fig. 11:**
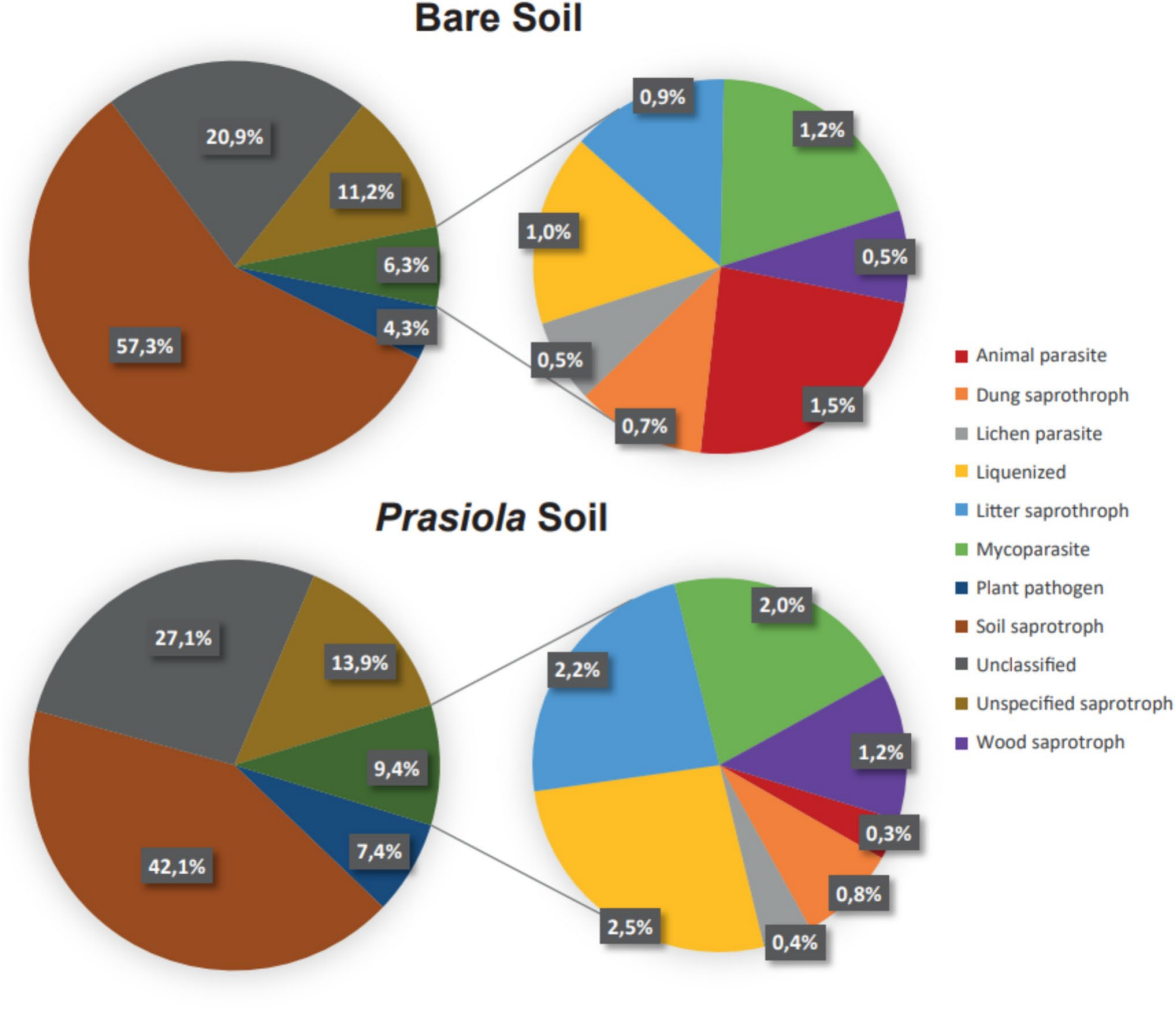
Relative abundance of different fungal functional categories assigned for two soil types based on data available in the Fungal Traits database

Regarding fungal communities, ecological functions were assigned to 92 out of the 174 fungal ASVs (~ 53%) that were classified at the genus level. These functions were grouped into a total of ten distinct categories, with an additional category for unclassified ASVs. Across both soil types, fungal saprotrophs were predominant, particularly soil saprotrophs (Fig. 11). Although statistical analyses did not reveal significant differences in functional categories between the two soil types, certain trends were observable (Table S9). Bare soils exhibited a higher abundance of ASVs assigned to soil saprotrophs, while lichenized fungi, plant pathogens, and wood saprotrophs were more abundant in the soils under *Prasiola* canopy (Fig. 11).

## Discussion

This study demonstrates that the establishment of a *Prasiola* canopy on Antarctic tundra soils influences not only their abiotic attributes but also the taxonomic composition of bacterial and fungal communities, leading to the replacement of taxa and their functional roles. In addition, the characterization of the bacterial microbiome associated with the *Prasiola* canopy revealed a taxonomically similar, yet structurally different, bacterial composition compared to the underlying soils.

### Taxonomic Characterization of *Prasiola* Species

The canopy analyzed in this study comprised multiple *Prasiola* species, with *P. crispa* and *P. antarctica* often occurring together. *P. crispa* often thrives in soils enriched by bird guano [30, 79], and in this case, the canopy was indeed developing near penguin rookeries and petrel nests. Recent studies have highlighted *P. antarctica*’s high plasticity and ability to thrive in harsh Antarctic environments, such as dry sandy soils near beaches or rocky beds beneath ephemeral freshwater streams [11, 30]. However, other species such as *P. glacialis*, *P. furfuracea*, and an undescribed *Prasiola*, previously reported in Antarctica [30], were not found in the studied canopies, despite the known preference of *P. glacialis* for non-permanent freshwater habitats [30]. *Prasiola furfuracea* and the still undescribed *Prasiola* are well-defined morphologically, molecularly, and ecologically, as they are almost always found in symbiosis with the ascomycete fungus *Mastodia* [36, 80, 81]. Our work further underscores the importance of using molecular phylogenetics for accurately distinguish *Prasiola* species, especially in the absence of clear macroscopic differences [30]. The *tuf*A marker proved to be extremely useful in these analyses [38].

### Effects of Canopy Establishment on Soil Properties

The results indicate that soil attributes, such as pH and organic matter, carbon, and nitrogen contents, are influenced by the establishment of *Prasiola* canopies, consistent with previous studies on Antarctic cryptogamic covers [26, 82–84]. Furthermore, dbRDA ordination suggests that variations in these abiotic factors play a key role in shaping the microbial community structure of the underlying soils, aligning with observations in other polar cryptogamic covers [27, 59, 85]. Moreover, canopy establishment can influence soil properties, such as water retention and temperature regimes, which in turn can affect microbial community structure [85–88]. While this effect has been extensively studied in covers dominated by lichens and mosses, both in situ and under laboratory conditions [86, 89–91], it has been scarcely explored for macroalgal canopies. Given the highly specific functional traits of various cryptogamic covers [92], further investigation is needed into how this particular *Prasiola* canopy modifies soil temperature and water fluxes. Such studies could also shed light on its potential impact on the taxonomic and functional diversity of microbial communities in these covered soils.

### Microbial Community Structure of Soils and* Prasiola* Canopy

The bacterial community structure within the *Prasiola* canopy was distinct from that of the underlying soil, resembling more closely the structure found in other cryptogamic covers [27, 93]. However, the dominant bacterial groups were largely the same in both the canopy and the soil beneath, with Bacteroidota being the most abundant phylum.

The bacterial community exhibited significant structural differences between soils with and without *Prasiola* canopy, revealing a clear impact of canopy establishment on the soil microbiome. In contrast to the soil beneath *Prasiola* canopy, Actinobacteriota predominated in bare soils, aligning with soil profiles from other cold regions, such as Antarctic, Arctic, and alpine environments [60, 94, 95]. Indeed, Actinobacteriota has been more commonly associated with soils having a low cryptogam coverage in Antarctica [96]. The relative abundance of the dominant phylum Bacteroidota in *Prasiola*-covered soil varies across different polar edaphic ecosystems. While this phylum predominates in some soils [95, 97, 98], it appears in lower abundances in others [99, 100], suggesting sensitivity to specific environmental factors. Additionally, recent research has indicated a preference for alkaline soils by Bacteroidota in Antarctica [101], which is consistent with our finding of increased abundance of these bacteria in the soils with higher pH beneath the *Prasiola* canopy.

In addition to Bacteroidota, other bacterial phyla as Gammaproteobacteria and Myxococcota showed higher abundances in soils under *Prasiola* canopies compared to bare soils. Increased abundances of Bacteroidota and Myxococcota have previously been reported in soils beneath other cryptogamic canopies compared to bare soils [27, 102], and their association with avian activity has been previously noted [103, 104]. Bacteroidota includes taxa capable of breaking down biomacromolecules [97, 105, 106], which could give them an advantage in colonizing soils with higher organic matter content, such as those beneath cryptogamic cover. Similarly, the saprotrophic ability of some members of Myxococcota [107] may also be favoured by the increased organic matter content under the *Prasiola* canopy. Interestingly, in contrast to this study, Gammaproteobacteria have not been reported to increase under plant and bryophyte canopies [27, 108]. Since the influence of cryptogamic canopies on soil microbial communities can depend on the specific cryptogam involved [26, 92], the observed effects in Gammaproteobacteria may be specific to the analyzed terrestrial macroalgae, which benefits this phylum known to perform a high variety of ecological functions [109]. On the other hand, the abundance of other taxa, such as WPS2 (candidatus Eremiobacterota) [110], decreased following canopy establishment. Eremiobacterota includes microorganisms with diverse metabolic capabilities and is particularly abundant in poorly developed soils under polar climates [111], aligning perfectly with the findings of our study.

Fungal profiles exhibited much more variability than bacterial ones, suggesting that random dispersal or unstudied local environmental factors have a stronger influence on fungal communities than canopy establishment. This aligns with previous findings by Brown and Jumpponen [94] regarding plant covers and by Ortiz-Rivero et al. [27] on bryophyte covers. The variability appears to increase under the macroalgal canopy, likely due to the formation of specific microenvironments at the interface with the cryptogamic cover [59, 94, 112–114]. Contrary to bacterial communities, the highest number of unique fungal ASVs was found in soils beneath the canopy. This difference may be attributed to an increase in *Prasiola*-specific fungal saprotrophs that benefit from the elevated organic matter resulting from canopy establishment [115, 116].

### Microbial Diversity

Contrasting patterns in the impact of cryptogamic cover development on soil microbial diversity have been reported, varying based on the dominant components of the cover [26, 27, 94, 117, 118]. For soils with and without *Prasiola* canopy, no significant differences in α-diversity were detected. However, despite the similarity in dominant taxa, the canopy exhibited significantly lower bacterial α-diversity compared to both types of soils, with only a small number of unique ASVs. It is well established that soils possess high complexity in their physical organization and chemical composition, resulting in the formation of microhabitats and microenvironments that support specific microorganisms [119]. These findings suggest that the macroalga itself does not introduce specific bacterial taxa into the underlying soil. Instead, the number of unique ASVs was higher in bare soils than beneath the *Prasiola* canopy. The canopy appears to select for and enrich specific soil microbial taxa, alongside a significantly higher abundance of taxonomically unclassified ASVs that may be habitat-specific [28, 120–122]. Future studies should focus on evaluating the role of these specific epi- or endophytic bacteria in the *Prasiola*’s physiology, growth, stress tolerance, and defence.

The high variability in Pielou’s evenness and Simpson and Shannon indexes for fungal communities within *Prasiola*-covered soils may be associated with the patchy distribution of angiosperms and bryophytes in the area, due to host-specific relationships established between fungal taxa and plants [123]. Moreover, colonization alters microbial community structure by increasing soil carbon and nitrogen in the surrounding environment [124]. In fact, the variability in soil attributes was greater in *Prasiola*-covered soils compared to bare soils. This spatial heterogeneity of soil physicochemical properties could also explain the observed trend of lower Simpson and Pielou’s evenness values in *Prasiola*-covered soils for fungal communities.

### Functional Bacterial and Fungal Soil Community Structure

Differences in bacterial functional roles between covered and bare soils were also detected. Bare soils showed a statistically significant higher potential for methanogenesis and autotrophic CO_2_ fixation compared to covered soils, with these two activities being almost negligible in the *Prasiola* canopy itself. The higher potential for autotrophic CO_2_ fixation in bare soils may be related to the fact that autotrophic microorganisms are pioneers in soil development in polar areas [125, 126]. As biological succession progresses and ecosystem functions diversify, the abundance of these autotrophs tends to decrease [59, 127, 128]. Moreover, the observed elevated potential for autotrophic CO_2_ fixation in bare soils could also be attributed to the higher abundance of phyla such as the Chloroflexi or the Alphaproteobacteria, both of which contain numerous photoautotrophic organisms [129, 130]. However, chemoautotrophic members of Actinobacteriota and Proteobacteria may also contribute to CO_2_ fixation [131]. The higher abundance of methanogens in bare soils may be associated with the predominance of CO_2_-fixing microorganisms under the cover, as they reduce CO_2_ during the methanogenic cycle [132]. The functional results for methanogenesis may be mainly explained by the abundance of Actinobacteriota and, especially, Alphaproteobacteria, as these two phyla are associated with high methanogenic potential [133–135]. Indeed, the highest potential for autotrophic CO_2_ fixation in sample APH5 and for methanogenesis in APH3 corresponds to the highest relative abundances of Actinobacteriota and Alphaproteobacteria, respectively.

As expected in an ecosystem with limited vascular plants, the most represented functional category within the fungal community was soil saprotrophism [59, 115, 136]. However, the establishment of the *Prasiola* canopy promoted an increased relative abundance of other ecosystem functions, potentially driven by changes in soil attributes, such as higher organic matter content [24, 137, 138]. This shift is reflected in the reduced abundance of soil saprotrophic taxa, such as Thelebolales or Mortierellales, in soils under the canopy, alongside a higher abundance of plant-dependent taxa, such as Xylariales or Helotiales [78]. The higher abundance of animal parasites in bare soil may be explained by the easier access for animals to these areas compared to vegetated ones, although the influence of soil attributes cannot be ruled out. Animal parasites are key components of the Antarctic biodiversity and are integral to ecosystem interactions [6, 139]. Our findings underscore the importance of considering secondary interactions, such as those among soil fauna, vegetation, and microbiota, alongside with direct effects of *Prasiola* on soil attributes, when studying changes in soil microbial communities.

Considering the specific impact of *Prasiola* canopies on soil microbial communities, it is essential to analyze the effects of various types of covers under different Antarctic environmental conditions. This understanding is crucial for anticipating future vegetation dynamics in deglaciated polar regions, especially as rising temperatures are expected to promote the greening of ice-free soils, including the expansion of cryptogamic covers in maritime-influenced areas [4, 140].

## Conclusions

The establishment of a canopy dominated by different species of the foliose macroalga *Prasiola* on Antarctic tundra soils, along with the associated transformation of their soil attributes, is linked to changes in the taxonomic and functional structure of bacterial and fungal communities. This study underscores the importance of characterizing the establishment of various cryptogamic canopy types to fully comprehend the evolution of Antarctic terrestrial ecosystems amidst the current accelerated climate change scenario.

## Supplementary Information

Below is the link to the electronic supplementary material.

Supplementary file1 (DOCX 998 KB)

## Data Availability

Molecular data uploading to Genbank repository is in process and ID numbers will be added in future revisions.
