# Supplementary material for: The Establishment of a Terrestrial Macroalga Canopy Impacts Microbial Soil Communities in Antarctica
Source: Microb Ecol. 2025 Feb 13;88(1):4. doi: 10.1007/s00248-025-02501-8 (PMC11825648; doi:10.1007/s00248-025-02501-8)
Supplement: Supplementary file 1 — Supplementary file1 (DOCX 998 KB) [file 248_2025_2501_MOESM1_ESM.docx]

| **Table S1** Sequences used in the phylogenetic analysis in this study. Newly generated sequences from this study are indicated with an asterisk (*). | | |
| --- | --- | --- |
| GenBank Number | Genbank species | Clade |
| HQ610263.141-791 | *Prasiola* sp. | *Prasiola delicata* |
| HQ610263.143-773 | *Prasiola* sp. | *Prasiola delicata* |
| HQ610267.1 | *Prasiola stipitata* | *Prasiola stipitata* |
| PQ998985  * | *Prasiola crispa* | *Prasiola crispa* |
| PQ998986  * | *Prasiola antarctica* | *Prasiola antarctica* |
| PQ998987  * | *Prasiola crispa* | *Prasiola crispa* |
| PQ998988  * | *Prasiola crispa* | *Prasiola crispa* |
| PQ998989  * | *Prasiola crispa* | *Prasiola crispa* |
| PQ998990  * | *Prasiola antarctica* | *Prasiola antarctica* |
| KF993432.1116-638 | *Rosenvingiella constricta* | *Rosenvingiella constricta* |
| KF993433.11-595 | *Prasiola meridionalis* | *Prasiola meridionalis* |
| KF993434.11-535 | *Prasiola meridionalis* | *Prasiola meridionalis* |
| KF993436.1110-632 | *Rosenvingiella radicans* | *Rosenvingiella radicans* |
| KF993439.11-687 | *Prasiola meridionalis* | *Prasiola meridionalis* |
| KF993440.11-749 | *Prasiola novaezelandiae* | *Prasiola novaezelandiae* |
| KF993440.124-733 | *Prasiola novaezelandiae* | *Prasiola novaezelandiae* |
| KF993440.13-733 | *Prasiola novaezelandiae* | *Prasiola novaezelandiae* |
| KF993442.11-733 | *Prasiola meridionalis* | *Prasiola meridionalis* |
| KF993442.13-741 | *Prasiola meridionalis* | *Prasiola meridionalis* |
| KF993443.11-698 | *Prasiola meridionalis* | *Prasiola meridionalis* |
| KF993445.1 | *Prasiola yunnanica* | *Prasiola japonica* |
| KF993446.1 | *Prasiola stipitata* | *Prasiola stipitata* |
| KF993446.11-733 | *Prasiola stipitata* | *Prasiola stipitata* |
| KF993447.1 | *Prasiola anctartica* | *Prasiola anctartica* |
| KF993447.11-722 | *Prasiola anctartica* | *Prasiola anctartica* |
| KF993447.11-738 | *Prasiola anctartica* | *Prasiola anctartica* |
| KF993447.113-722 | *Prasiola anctartica* | *Prasiola anctartica* |
| KF993449.11-623 | *Prasiola calophylla* | *Prasiola calophylla* |
| KF993450.11-700 | *Prasiola crispa* | *Prasiola crispa* |
| KF993450.177-633 | *Prasiola crispa* | *Prasiola crispa* |
| KF993451.11-511 | *Prasiola stipitata* | *Prasiola stipitata* |
| KF993452.11-632 | *Prasiola stipitata* | *Prasiola stipitata* |
| KF993454.11-751 | *Prasiola* cf. *delicata* | *Prasiola delicata* |
| KF993455.11-700 | *Prasiola calophylla* | *Prasiola calophylla* |
| KF993456.11-710 | *Prasiola furfuracea* | *Prasiola furfuracea* |
| KR017748.171290-71999 | *Prasiola crispa* | *Prasiola crispa* |
| KR261679.127-759 | *Prasiola japonica* | *Prasiola japonica* |
| KR261682.127-759 | *Prasiola japonica* | *Prasiola japonica* |
| KR261685.150-759 | *Prasiola japonica* | *Prasiola japonica* |
| KT354069.130-762 | *Prasiola* cf. *meridionalis* | *Prasiola meridionalis* |
| KT354069.132-780 | *Prasiola* cf. *meridionalis* | *Prasiola meridionalis* |
| KT354070.132-760 | *Prasiola delicata* | *Prasiola delicata* |
| KT354072.140-772 | *Prasiola* cf. *meridionalis* | *Prasiola meridionalis* |
| KT354073.11-739 | *Prasiola japonica* | *Prasiola japonica* |
| KT354075.136-768 | *Prasiola japonica* | *Prasiola japonica* |
| KT354075.159-768 | *Prasiola japonica* | *Prasiola japonica* |
| KT355706.182-814 | *Prasiola japonica* | *Prasiola japonica* |
| KY028915.11-574 | *Prasiola borealis* | *Prasiola furfuracea* |
| KY028930.11-574 | *Prasiola borealis* | *Prasiola furfuracea* |
| KY029019.11-574 | *Prasiola borealis* | *Prasiola furfuracea* |
| KY028956.11-574 | *Prasiola borealis* | *Prasiola furfuracea* |
| KF993441.11-733 | *Prasiola borealis* | *Prasiola furfuracea* |
| KF993441.13-751 | *Prasiola borealis* | *Prasiola furfuracea* |
| KY028957.11-574 | *Prasiola borealis* | *Prasiola furfuracea* |
| KY028958.1 | *Prasiola borealis* | *Prasiola furfuracea* |
| KY028961.11-574 | *Prasiola borealis* | *Prasiola furfuracea* |
| KY028964.1 | *Prasiola borealis* | *Prasiola furfuracea* |
| KY028964.11-574 | *Prasiola borealis* | *Prasiola furfuracea* |
| MN184611.141-773 | *Prasiola furfuracea* | *Prasiola furfuracea* |
| MN184611.143-791 | *Prasiola furfuracea* | *Prasiola furfuracea* |
| MN184611.164-773 | *Prasiola furfuracea* | *Prasiola furfuracea* |
| MH571169.1110-819 | *Prasiola furfuracea* | *Prasiola furfuracea* |
| MH571169.1182-755 | *Prasiola furfuracea* | *Prasiola furfuracea* |
| MH571169.187-819 | *Prasiola furfuracea* | *Prasiola furfuracea* |
| MH571169.187-836 | *Prasiola furfuracea* | *Prasiola furfuracea* |
| LN877826.11-750 | *Prasiola furfuracea* | *Prasiola furfuracea* |
| LN877826.124-733 | *Prasiola furfuracea* | *Prasiola furfuracea* |
| KY028970.11-574 | *Prasiola* sp. | *Prasiola* sp. |
| KY028978.11-574 | *Prasiola* sp. | *Prasiola* sp. |
| KY028986.11-574 | *Prasiola* sp. | *Prasiola* sp. |
| KY028992.11-574 | *Prasiola* sp. | *Prasiola* sp. |
| KY028995.11-574 | *Prasiola* sp. | *Prasiola* sp. |
| KY029002.11-574 | *Prasiola* sp. | *Prasiola* sp. |
| KY029006.11-574 | *Prasiola* sp. | *Prasiola* sp. |
| KY029009.11-574 | *Prasiola* sp. | *Prasiola* sp. |
| KY029013.11-574 | *Prasiola* sp. | *Prasiola* sp. |
| KY029015.11-574 | *Prasiola* sp. | *Prasiola* sp. |
| KY029017.11-574 | *Prasiola* sp. | *Prasiola* sp. |
| KY029018.11-574 | *Prasiola* sp. | *Prasiola* sp. |
| KY029020.11-566 | *Prasiola delicata* | *Prasiola delicata* |
| LN877821.11-733 | *Prasiola crispa* | *Prasiola crispa* |
| LN877821.1109-665 | *Prasiola crispa* | *Prasiola crispa* |
| LN877821.124-733 | *Prasiola crispa* | *Prasiola crispa* |
| LN877830.1143-665 | *Rosenvingiella australis* | *Rosenvingiella australis* |
| LN877834.1 | *Rosenvingiella radicans* | *Rosenvingiella radicans* |
| LN877837.1143-665 | *Rosenvingiella constricta* | *Rosenvingiella constricta* |
| MF347444.1221-743 | *Rosenvingiella australis* | *Rosenvingiella australis* |
| MF347445.1195-717 | *Rosenvingiellopsis constricta* | *Rosenvingiella constricta* |
| MN145932.1 | *Rosenvingiella radicans* | *Prasiola crispa* |
| MN145933.135-767 | *Prasiola crispa* | *Prasiola crispa* |
| MN145933.158-767 | *Prasiola crispa* | *Prasiola crispa* |
| MN145934.1143-699 | *Prasiola crispa* | *Prasiola crispa* |
| MN145935.1 | *Prasiola crispa* subsp*. antarctica* | *Prasiola anctartica* |
| MN145935.135-767 | *Prasiola crispa* subsp*. antarctica* | *Prasiola anctartica* |
| MN145935.158-767 | *Prasiola crispa* subsp*. antarctica* | *Prasiola anctartica* |
| MZ198522.11-717 | *Prasiola* sp. | *Prasiola furfuracea* |
| MZ198522.18-717 | *Prasiola* sp. | *Prasiola furfuracea* |

**Fig. S1** FastQC histograms displaying the mean quality scores of the 16S and ITS markers, representing bacterial and fungal communities, respectively.

| **Table S2** Mean values and standard deviations of measured soil attributes. Asterisks indicate statistical significance as determined by the Kruskal-Wallis test. | | | | | |
| --- | --- | --- | --- | --- | --- |
| Sample type | N content (%) | C content (%) | C/N | M.O (%) | pH |
| Bare soil | 0.12 ± 0.049 | 0.5 ± 0.25 | 3.89 ± 0.47 | 4.11 ± 1.02 | 4.98 ± 0.09 |
| *Prasiola*-covered soil | 0.2 ± 0.09 | 1.12 ± 0.76 | 5.17 ± 1.17 | 7.21 ± 3.08 | 5.06 ± 0.119 |
| P value (ANOVA or Kruskal-Wallis) | 0.09524* | 0.1183 | 0.08088 | 0.05556* | 0.1716 |

**Fig. S2** Relative abundance of bacterial phyla and fungal orders at individual sampling points, as revealed by high-throughput Illumina sequencing. The category 'Others' includes groups representing less than 0.25% of the total relative abundance in bacteria and less than 0.005% in fungi

**Table S3** P-Values from the Kruskal-Wallis test on the relative abundances of bacterial phyla across different sample types. Statistically significant differences (P-values < 0.05) are highlighted in bold.

| Phylum | Bare soil/  *Prasiola*-covered Soil | Canopy/  *Prasiola-*covered soil |
| --- | --- | --- |
| *Bacteroidota* | **0.01431** | 0.0758 |
| *Alphaproteobacteria* | 0.08641 | **0.009023** |
| *Gammaproteobacteria* | **0.01431** | 0.2506 |
| *Actinobacteriota* | **0.02749** | **0.009023** |
| *Chloroflexi* | 0.1416 | **0.01629** |
| *Unclassified* | 0.08641 | **0.009023** |
| *Cyanobacteria* | 0.3272 | 0.9168 |
| *Myxococcota* | **0.02749** | **0.02828** |
| *Gemmatimonadota* | 0.8065 | **0.009023** |
| WPS 2 | **0.01431** | **0.008208** |
| Others | 0.2207 | **0.009023** |

**Table S4** P-Values from the Kruskal-Wallis test on the relative abundance of fungal orders across different sample types. Statistically significant differences (P-values < 0.05) are highlighted in bold

| Order | Bare soil/  *Prasiola-*covered Soil |
| --- | --- |
| Mortierellales | 0.4624 |
| *Helotiales* | 0.8065 |
| *Thelebolales* | 0.2207 |
| *Hypocreales* | 0.3272 |
| *Tremellales* | 0.8065 |
| *Eurotiales* | 1 |
| *Cystofilobasidiales* | 0.3272 |
| *Pleosporales* | 0.4624 |
| *Xylariales* | 0.3272 |
| *Kriegeriales* | 0.08641 |
| *Verrucariales* | 0.2187 |
| Unclassified | 0.3272 |
| Others | 0.6242 |

| **Table S5** P-Values from the Kruskal-Wallis (KW) and the Mann-Whitney (MW) tests on *α-*diversity indices across different sample types. Statistically significant differences (P-values < 0.05) are highlighted in bold. | | | | | | | | |
| --- | --- | --- | --- | --- | --- | --- | --- | --- |
| Samples compared | Richness | | Shannon | | Simpson | | Pielou`s Evenness | |
|  | MW | KW | MW | KW | MW | KW | MW | KW |
| Bare soil /  *Prasiola*-covered soil | 0.111 | **0.006** | 0.730 | **0.011** | 0.905 | **0.016** | 0.556 | **0.010** |
| *Prasiola*-covered soil / *Prasiola* canopy | **0.008** |  | **0.008** |  | **0.008** |  | **0.024** |  |

| **Table S6** P-Values obtained from Mann-Whitney test on fungal *α-*diversity indices across different sample types. Statistically significant differences (P-values < 0.05) are highlighted in bold. | | | | |
| --- | --- | --- | --- | --- |
| Samples compared | Richness | Shannon | Simpson | Pielou`s Evenness |
| Bare soil /  *Prasiola*-covered soil | 0.623 | 0.413 | 0.111 | 0.11 |

| **Table S7** P-Values from Aldex2 Welch’s test from the 405 bacterial pathways identified with Picrust2 across different sample types. Statistically significant differences (P-values < 0.05) are highlighted in bold. | | |
| --- | --- | --- |
| Bacterial pathway | Bare soil/ *Prasiola*-covered soil | *Prasiola*-covered soil/  *Prasiola* canopy |
| methanol oxidation to carbon dioxide | **0.03865178** | 0.5969715 |
| superpathway of glycerol degradation to 1,3-propanediol | 0.13583844 | 0.63613423 |
| superpathway of glycol metabolism and degradation | 0.05068929 | 0.55429462 |
| superpathway of methylglyoxal degradation | **0.03611944** | **0.00467289** |
| ectoine biosynthesis | 0.81668535 | 0.09553101 |
| norspermidine biosynthesis | **0.01892124** | 0.80843312 |
| superpathway of arginine and polyamine biosynthesis | 0.91286303 | **0.00275248** |
| superpathway of polyamine biosynthesis I | 0.56325552 | **0.0023023** |
| superpathway of polyamine biosynthesis II | 0.49652214 | **0.00207506** |
| creatinine degradation I | 0.3936048 | 0.47935595 |
| creatinine degradation II | **0.00357257** | 1 |
| glycine betaine degradation I | 0.07196836 | 0.13232456 |
| aromatic biogenic amine degradation (bacteria) | 0.4210417 | 0.0854358 |
| superpathway of N-acetylglucosamine N-acetylmannosamine and N-acetylneuraminate degradation | 0.16455969 | 0.38095255 |
| superpathway of ornithine degradation | 0.573538 | 0.34872361 |
| allantoin degradation IV (anaerobic) | 0.64716133 | 0.57525394 |
| allantoin degradation to glyoxylate III | 0.32755143 | 0.58151285 |
| L-histidine biosynthesis | 0.49105233 | **0.00298571** |
| L-isoleucine biosynthesis I (from threonine) | 0.3890209 | **0.0029365** |
| L-isoleucine biosynthesis II | 0.39841137 | **0.00294033** |
| L-isoleucine biosynthesis III | 0.29620231 | **0.00296689** |
| L-isoleucine biosynthesis IV | 0.82521782 | **0.00382551** |
| L-lysine biosynthesis I | 0.320881 | **0.02297682** |
| L-lysine biosynthesis II | 0.27279 | 0.06169665 |
| L-lysine biosynthesis III | 0.60064562 | **0.00296304** |
| L-lysine biosynthesis VI | 0.64373022 | **0.0029674** |
| L-methionine biosynthesis I | 0.13486035 | 0.08165886 |
| L-methionine biosynthesis III | 0.67320278 | **0.00291221** |
| L-methionine salvage cycle III | 0.29247334 | 0.22921085 |
| L-ornithine biosynthesis | 0.20178703 | **0.00294754** |
| L-tryptophan biosynthesis | 0.37592495 | **0.00302351** |
| L-valine biosynthesis | 0.38960668 | **0.00293719** |
| superpathway of aromatic amino acid biosynthesis | 0.31131449 | **0.00344508** |
| superpathway of branched amino acid biosynthesis | 0.28767649 | **0.00296743** |
| superpathway of L-alanine biosynthesis | 0.63350469 | **0.00399102** |
| superpathway of L-aspartate and L-asparagine biosynthesis | 0.33408464 | 0.07963833 |
| superpathway of L-isoleucine biosynthesis I | 0.61739448 | **0.00296822** |
| superpathway of L-lysine, L-threonine and L-methionine biosynthesis I | 0.24771503 | **0.01505486** |
| superpathway of L-methionine biosynthesis (by sulfhydrylation) | 0.6735807 | **0.00295884** |
| superpathway of L-methionine biosynthesis (transsulfuration) | 0.43991253 | **0.00944647** |
| superpathway of L-phenylalanine biosynthesis | **0.02423104** | **0.00286141** |
| superpathway of L-serine and glycine biosynthesis I | 0.44271951 | **0.003118** |
| superpathway of L-threonine biosynthesis | 0.89948796 | **0.00297539** |
| superpathway of L-tryptophan biosynthesis | 0.70795313 | 0.62794754 |
| superpathway of L-tyrosine biosynthesis | **0.03210373** | **0.00295626** |
| beta-alanine biosynthesis II | 0.08079192 | 0.19140759 |
| L-arginine biosynthesis I (via L-ornithine) | 0.37407466 | **0.00294151** |
| L-arginine biosynthesis II (acetyl cycle) | **0.04597445** | **0.01341759** |
| L-arginine biosynthesis III (via N-acetyl-L-citrulline) | 0.8724868 | **0.00272603** |
| L-arginine biosynthesis IV (archaebacteria) | 0.37850556 | **0.00294204** |
| L-arginine degradation II (AST pathway) | **0.01822796** | 0.20867666 |
| L-glutamate and L-glutamine biosynthesis | 0.8461317 | **0.00328552** |
| L-histidine degradation I | 0.75036653 | **0.00359559** |
| L-histidine degradation II | **0.00388806** | **0.00272832** |
| L-leucine degradation I | 0.2528952 | **0.0041543** |
| L-tryptophan degradation IX | 0.14149859 | **0.02961939** |
| L-tryptophan degradation to 2-amino-3-carboxymuconate semialdehyde | **0.00878739** | **0.00438987** |
| L-tryptophan degradation XII (Geobacillus) | 0.11140775 | 0.08983199 |
| L-tyrosine degradation I | 0.05602907 | 0.00979709 |
| L-valine degradation I | 0.27604696 | 0.46108684 |
| superpathway of L-arginine and L-ornithine degradation | 0.68229642 | 0.60896555 |
| superpathway of L-arginine, putrescine, and 4-aminobutanoate degradation | 0.64638715 | 0.61373185 |
| superpathway of L-threonine metabolism | 0.70630165 | 0.58660376 |
| methanogenesis from acetate | 0.10170151 | 0.06080527 |
| 3-phenylpropanoate and 3-(3-hydroxyphenyl)propanoate degradation to 2-oxopent-4-enoate | **0.02995005** | 0.22152877 |
| 3-phenylpropanoate degradation | 0.51939174 | **0.00274849** |
| 4-coumarate degradation (anaerobic) | 0.34341697 | 0.52104947 |
| chorismate biosynthesis from 3-dehydroquinate | 0.41413227 | **0.00297729** |
| chorismate biosynthesis I | 0.28864543 | **0.00350321** |
| gallate degradation I | **0.00080753** | **0.00235104** |
| gallate degradation II | **0.00096423** | **0.00355415** |
| 2-aminophenol degradation | 0.29196674 | **0.00334206** |
| 3-phenylpropanoate and 3-(3-hydroxyphenyl)propanoate degradation | **0.0204571** | 0.30615854 |
| 4-hydroxyacetophenone degradation | 0.25806538 | 0.34542002 |
| 4-hydroxyphenylacetate degradation | **0.00547022** | **0.04825977** |
| 4-methylcatechol degradation (ortho cleavage) | 0.4198853 | 0.07091205 |
| cinnamate and 3-hydroxycinnamate degradation to 2-oxopent-4-enoate | **0.02916162** | 0.21606392 |
| mandelate degradation I | **0.02912911** | **0.02000305** |
| mandelate degradation to acetyl-CoA | 0.08864653 | **0.00716642** |
| methylgallate degradation | **0.00078189** | **0.0024103** |
| nicotinate degradation I | **0.02088081** | 0.10831079 |
| phenylacetate degradation I (aerobic) | 0.56939535 | **0.03808331** |
| protocatechuate degradation I (meta-cleavage pathway) | **0.00058894** | **0.00238551** |
| protocatechuate degradation II (ortho-cleavage pathway) | 0.12447608 | **0.00304133** |
| superpathway of aerobic toluene degradation | 0.80302836 | **0.00518709** |
| superpathway of phenylethylamine degradation | 0.24764509 | **0.00819063** |
| superpathway of salicylate degradation | **0.02145087** | **0.00167848** |
| superpathway of vanillin and vanillate degradation | **0.00176986** | 0.88529424 |
| syringate degradation | **0.00163385** | **0.00264933** |
| toluene degradation I (aerobic) (via o-cresol) | **0.03208962** | 0.22261531 |
| toluene degradation II (aerobic) (via 4-methylcatechol) | **0.03234332** | 0.22021498 |
| toluene degradation III (aerobic) (via p-cresol) | 0.71239678 | 0.06652487 |
| toluene degradation IV (aerobic) (via catechol) | 0.63629484 | 0.08476436 |
| vanillin and vanillate degradation I | **0.00177168** | 0.88458065 |
| vanillin and vanillate degradation II | **0.00166865** | 0.87370026 |
| 2-nitrobenzoate degradation I | **0.01728813** | 0.06864934 |
| aromatic compounds degradation via beta-ketoadipate | **0.04879418** | **0.00165655** |
| benzoyl-CoA degradation I (aerobic) | **0.00087192** | 0.13074125 |
| catechol degradation I (meta-cleavage pathway) | **0.01564322** | 0.31868772 |
| catechol degradation II (meta-cleavage pathway) | **0.04312533** | 0.05840237 |
| catechol degradation III (ortho-cleavage pathway) | **0.04844175** | **0.00165624** |
| catechol degradation to beta-ketoadipate | **0.01432569** | **0.00098592** |
| catechol degradation to 2-oxopent-4-enoate II | **0.00611286** | **0.02004896** |
| meta cleavage pathway of aromatic compounds | 0.92897121 | 0.11666327 |
| chlorosalicylate degradation | 0.84444358 | 0.1347854 |
| Calvin-Benson-Bassham cycle | 0.42227899 | **0.00310036** |
| glyoxylate assimilation | 0.2317543 | 0.16576762 |
| incomplete reductive TCA cycle | 0.3517287 | 0.33971621 |
| 3-hydroxypropanoate cycle | 0.19595537 | 0.17632864 |
| reductive acetyl coenzyme A pathway | **0.01108729** | 0.08575134 |
| reductive TCA cycle I | **0.04634195** | 0.42550362 |
| reductive TCA cycle II | **0.01353991** | **0.00303901** |
| superpathway of the 3-hydroxypropanoate cycle | 0.22083719 | 0.17180689 |
| superpathway of C1 compounds oxidation to CO2 | 0.50141342 | 0.66590946 |
| formaldehyde assimilation I (serine pathway) | 0.26813288 | 0.20027074 |
| formaldehyde assimilation II (RuMP Cycle) | 0.0838577 | **0.00245279** |
| formaldehyde oxidation I | 0.0836823 | **0.00245377** |
| colanic acid building blocks biosynthesis | 0.72167001 | **0.01084039** |
| 2-amino-3-carboxymuconate semialdehyde degradation to 2-oxopentenoate | **0.01725062** | 0.12853412 |
| glutaryl-CoA degradation | 0.53544221 | 0.64055782 |
| superpathway of N-acetylneuraminate degradation | **0.04069506** | 0.19948558 |
| 2-methylcitrate cycle I | **0.01475677** | 0.89685421 |
| 2-methylcitrate cycle II | **0.01459076** | **0.00354783** |
| coenzyme A biosynthesis I | 0.39203352 | **0.0029744** |
| pantothenate and coenzyme A biosynthesis I | 0.4319903 | **0.00296684** |
| phosphopantothenate biosynthesis I | 0.32701618 | **0.00312877** |
| mono-trans, poly-cis decaprenyl phosphate biosynthesis | **0.03573329** | 0.62731399 |
| coenzyme M biosynthesis I | 0.49619676 | 0.11248777 |
| flavin biosynthesis I (bacteria and plants) | 0.40738064 | **0.00297138** |
| N10-formyl-tetrahydrofolate biosynthesis | 0.49458451 | **0.00298743** |
| superpathway of tetrahydrofolate biosynthesis | 0.90437959 | **0.00296822** |
| superpathway of tetrahydrofolate biosynthesis and salvage | 0.86464551 | **0.00297437** |
| mycolyl-arabinogalactan-peptidoglycan complex biosynthesis | **0.00127413** | 0.92480998 |
| peptidoglycan biosynthesis I (meso-diaminopimelate containing) | 0.33437557 | **0.00308481** |
| peptidoglycan biosynthesis II (staphylococci) | 0.497516 | 1 |
| peptidoglycan biosynthesis III (mycobacteria) | 0.29872434 | **0.00299047** |
| peptidoglycan biosynthesis IV (Enterococcus faecium) | 0.7093803 | 0.24581933 |
| peptidoglycan biosynthesis V (beta-lactam resistance) | 0.28115315 | 1 |
| peptidoglycan maturation (meso-diaminopimelate containing) | **0.03752933** | **0.00439533** |
| superpathway of mycolyl-arabinogalactan-peptidoglycan complex biosynthesis | **0.00193066** | 0.48343446 |
| UDP-N-acetylmuramoyl-pentapeptide biosynthesis I (meso-diaminopimelate containing) | 0.3259015 | **0.00314763** |
| UDP-N-acetylmuramoyl-pentapeptide biosynthesis II (lysine-containing) | 0.2909934 | **0.00298857** |
| teichoic acid (poly-glycerol) biosynthesis | 0.12619833 | 0.1002515 |
| chlorophyllide a biosynthesis I (aerobic light-dependent) | 0.29452858 | 0.6746981 |
| chlorophyllide a biosynthesis II (anaerobic) | 0.46421478 | 0.37584519 |
| chlorophyllide a biosynthesis III (aerobic light independent) | 0.46421477 | 0.37602159 |
| superpathway of bacteriochlorophyll a biosynthesis | 0.28335797 | 0.1939348 |
| vitamin B6 degradation | 0.11756733 | 0.26008382 |
| nylon-6 oligomer degradation | 0.68536917 | 0.1757519 |
| octane oxidation | 0.12806423 | **0.02092564** |
| coenzyme B biosynthesis | 0.92109261 | 0.37138507 |
| factor 420 biosynthesis | 0.9410739 | 0.38398321 |
| mycothiol biosynthesis | **0.01397661** | 0.38741716 |
| 1,4-dihydroxy-2-naphthoate biosynthesis I | **0.030088** | 0.15272915 |
| 1, 4-dihydroxy-6-naphthoate biosynthesis I | 0.15096211 | **0.00714493** |
| 1, 4-dihydroxy-6-naphthoate biosynthesis II | 0.41308234 | **0.00880853** |
| superpathway of demethylmenaquinol-6 biosynthesis I | **0.00326775** | 0.3904254 |
| superpathway of demethylmenaquinol-6 biosynthesis II | 0.67610002 | 0.60401168 |
| superpathway of demethylmenaquinol-8 biosynthesis | 0.05463563 | 0.09337166 |
| superpathway of demethylmenaquinol-9 biosynthesis | **0.00322741** | 0.39211403 |
| superpathway of menaquinol-10 biosynthesis | **0.00567539** | 0.35390524 |
| superpathway of menaquinol-11 biosynthesis | 0.06047965 | 0.08519291 |
| superpathway of menaquinol-12 biosynthesis | 0.06095618 | 0.08517348 |
| superpathway of menaquinol-13 biosynthesis | 0.0615029 | 0.08524874 |
| superpathway of menaquinol-6 biosynthesis I | **0.00569932** | 0.35296925 |
| superpathway of menaquinol-7 biosynthesis | **0.04509117** | 0.16220641 |
| superpathway of menaquinol-8 biosynthesis I | 0.07340668 | 0.07572328 |
| superpathway of menaquinol-8 biosynthesis II | 0.12555022 | 0.88289458 |
| superpathway of menaquinol-9 biosynthesis | **0.00562105** | 0.35246722 |
| superpathway of phylloquinol biosynthesis | **0.03123095** | 0.14565161 |
| superpathway of ubiquinol-8 biosynthesis (prokaryotic) | 0.18054488 | **0.0085204** |
| ubiquinol-10 biosynthesis (prokaryotic) | 0.1778482 | **0.01066532** |
| ubiquinol-7 biosynthesis (prokaryotic) | 0.17927135 | **0.01071149** |
| ubiquinol-8 biosynthesis (prokaryotic) | 0.178317 | **0.01068159** |
| ubiquinol-9 biosynthesis (prokaryotic) | 0.17772142 | **0.01066896** |
| vitamin E biosynthesis (tocopherols) | 0.7510536 | 0.21019854 |
| NAD biosynthesis I (from aspartate) | 0.23502309 | **0.00320508** |
| NAD biosynthesis II (from tryptophan) | **0.01175029** | **0.00348192** |
| NAD salvage pathway I | 0.14894769 | **0.0029324** |
| NAD salvage pathway II | **0.00031957** | **0.04718581** |
| biotin biosynthesis I | **0.004866** | **0.00292698** |
| biotin biosynthesis II | 0.05414896 | 0.40018386 |
| cob(II)yrinate a, c-diamide biosynthesis I (early cobalt insertion) | 0.58336924 | 0.90693509 |
| cob(II)yrinate a | 0.07766815 | **0.00549101** |
| S-adenosyl-L-methionine cycle I | 0.27115402 | **0.0028544** |
| adenosylcobalamin biosynthesis from cobyrinate a,c-diamide I | 0.53465291 | **0.00286318** |
| adenosylcobalamin biosynthesis I (early cobalt insertion) | **0.01598368** | 0.25560232 |
| adenosylcobalamin biosynthesis II (late cobalt incorporation) | 0.38866772 | 0.40413749 |
| adenosylcobalamin salvage from cobinamide I | 0.06877417 | **0.00289638** |
| adenosylcobalamin salvage from cobinamide II | 0.69382037 | **0.00288937** |
| heme biosynthesis I (aerobic) | 0.08616997 | **0.00296703** |
| heme biosynthesis II (anaerobic) | 0.89725907 | **0.0029717** |
| superpathay of heme biosynthesis from glutamate | 0.671016 | **0.00301049** |
| superpathway of heme biosynthesis from glycine | **0.04186734** | 0.15577251 |
| superpathway of heme biosynthesis from uroporphyrinogen-III | **0.0341636** | 0.17768013 |
| pyridoxal 5'-phosphate biosynthesis I | 0.17772572 | 0.45977465 |
| superpathway of pyridoxal 5'-phosphate biosynthesis and salvage | 0.1081892 | 0.41033695 |
| superpathway of thiamin diphosphate biosynthesis I | 0.36912009 | **0.00343952** |
| superpathway of thiamin diphosphate biosynthesis II | 0.10022699 | 0.07863962 |
| thiamin salvage II | 0.62136712 | **0.00296869** |
| thiazole biosynthesis I (E. coli) | **0.01484512** | **0.01126268** |
| thiazole biosynthesis II (Bacillus) | 0.10423352 | 0.07693154 |
| mycolate biosynthesis | **0.02138882** | **0.0030586** |
| superpathway of phospholipid biosynthesis I (bacteria) | 0.43530159 | **0.00285309** |
| phospholipases | 0.09064349 | 0.34758449 |
| sitosterol degradation to androstenedione | **0.04246083** | 0.48155458 |
| androstenedione degradation | 0.43380331 | 0.4174635 |
| (5Z)-dodec-5-enoate biosynthesis | **0.01813459** | **0.00310399** |
| cis-vaccenate biosynthesis | 0.36359059 | **0.00262877** |
| fatty acid elongation -- saturated | 0.80023256 | **0.00275167** |
| fatty acid salvage | 0.46256774 | **0.00334467** |
| gondoate biosynthesis (anaerobic) | 0.34651994 | **0.00244403** |
| oleate biosynthesis IV (anaerobic) | **0.02091907** | **0.00308051** |
| palmitate biosynthesis II (bacteria and plants) | **0.00534576** | 0.61007904 |
| palmitoleate biosynthesis I (from (5Z)-dodec-5-enoate) | **0.01737906** | **0.00331942** |
| stearate biosynthesis II (bacteria and plants) | **0.01767899** | **0.00339811** |
| superpathway of fatty acid biosynthesis initiation (E. coli) | **0.01468635** | **0.00416353** |
| fatty acid beta-oxidation I | 0.84226398 | **0.00296157** |
| acetylene degradation | **0.01931488** | 0.17565865 |
| heterolactic fermentation | **0.01706914** | 0.26703142 |
| hexitol fermentation to lactate, formate, ethanol and acetate | 0.06481051 | 0.16005925 |
| glycerol degradation to butanol | 0.59926506 | 0.07116095 |
| mixed acid fermentation | 0.30753395 | **0.00302846** |
| pyruvate fermentation to acetate and lactate II | 0.05319153 | 0.51297046 |
| pyruvate fermentation to acetone | **0.04228837** | 0.06342856 |
| pyruvate fermentation to butanoate | **0.03170547** | 0.20166635 |
| pyruvate fermentation to isobutanol (engineered) | 0.21625783 | **0.00347787** |
| pyruvate fermentation to propanoate I | 0.35028947 | **0.00304426** |
| superpathway of Clostridium acetobutylicum acidogenic fermentation | **0.03191201** | 0.19046282 |
| 1,3-propanediol biosynthesis (engineered) | 0.39189862 | 1 |
| homolactic fermentation | 0.35226634 | **0.00468331** |
| L-1,2-propanediol degradation | 0.12260759 | 0.43829255 |
| L-glutamate degradation V (via hydroxyglutarate) | 0.5068796 | 0.63570096 |
| L-glutamate degradation VIII (to propanoate) | 0.51660768 | 0.39025521 |
| 4-aminobutanoate degradation V | **0.03004167** | 0.39312357 |
| acetyl-CoA fermentation to butanoate II | 0.51142581 | **0.04180041** |
| Bifidobacterium shunt | **0.01281068** | 0.19700737 |
| L-lysine fermentation to acetate and butanoate | 0.19369756 | 0.12361385 |
| succinate fermentation to butanoate | 0.28101819 | 0.13562315 |
| purine nucleobases degradation I (anaerobic) | 0.23265151 | **0.00303735** |
| 6-hydroxymethyl-dihydropterin diphosphate biosynthesis I | 0.74035626 | **0.00297136** |
| 6-hydroxymethyl-dihydropterin diphosphate biosynthesis III (Chlamydia) | 0.62152212 | **0.00296861** |
| aerobic respiration I (cytochrome c) | 0.2142872 | **0.00298311** |
| ethylmalonyl-CoA pathway | **0.04742301** | 0.08473639 |
| glycolysis I (from glucose 6-phosphate) | 0.32518688 | **0.00451219** |
| glycolysis II (from fructose 6-phosphate) | 0.28546979 | **0.00767449** |
| glycolysis III (from glucose) | 0.47512388 | **0.00300497** |
| glycolysis V (Pyrococcus) | 0.71480252 | 0.31009964 |
| glyoxylate cycle | 0.40436898 | **0.02927402** |
| isopropanol biosynthesis | **0.04262929** | 0.06352832 |
| methyl ketone biosynthesis | 0.94733075 | **0.01760353** |
| methylaspartate cycle | 0.86533286 | 0.06675024 |
| pentose phosphate pathway | 0.38713554 | **0.00515123** |
| pentose phosphate pathway (non-oxidative branch) | 0.49654831 | **0.00297554** |
| superpathway of glycolysis and Entner-Doudoroff | 0.41261918 | **0.00435083** |
| superpathway of glycolysis, pyruvate dehydrogenase, TCA, and glyoxylate bypass | 0.76690513 | **0.00291657** |
| TCA cycle I (prokaryotic) | 0.47054983 | **0.00295948** |
| TCA cycle IV (2-oxoglutarate decarboxylase) | 0.46359896 | **0.00298282** |
| TCA cycle V (2-oxoglutarate:ferredoxin oxidoreductase) | 0.33888556 | **0.0033523** |
| TCA cycle VI (obligate autotrophs) | 0.76535411 | **0.00296961** |
| TCA cycle VII (acetate-producers) | **0.01890446** | **0.00273719** |
| TCA cycle VIII (helicobacter) | 0.51140537 | **0.02112223** |
| lipid IVA biosynthesis | 0.15400372 | **0.00290212** |
| polymyxin resistance | 0.75038343 | 0.73108162 |
| superpathway of (Kdo)2-lipid A biosynthesis | 0.62157781 | 0.63937178 |
| superpathway of lipopolysaccharide biosynthesis | 0.65479199 | 0.66051476 |
| Kdo transfer to lipid IVA III (Chlamydia) | 0.13066186 | **0.00287967** |
| ppGpp biosynthesis | 0.35268803 | **0.01477806** |
| mannosylglycerate biosynthesis I | 0.06752392 | 0.26500773 |
| urea cycle | 0.09251664 | 0.22957088 |
| nitrate reduction I (denitrification) | **0.03108888** | 0.25660485 |
| nitrate reduction VI (assimilatory) | **0.02154734** | **0.01345363** |
| nitrifier denitrification | 0.10444544 | 0.15857105 |
| tRNA charging | 0.85827315 | **0.00302241** |
| tRNA processing | 0.76134868 | **0.00634853** |
| queuosine biosynthesis | 0.34425094 | **0.00316582** |
| S-methyl-5-thio-alpha-D-ribose 1-phosphate degradation | 0.2844714 | 0.23080231 |
| superpathway of purine deoxyribonucleosides degradation | 0.098488 | **0.00522431** |
| 8-amino-7-oxononanoate biosynthesis I | **0.00253509** | **0.0034924** |
| superpathway of (R, R)-butanediol biosynthesis | 0.49265778 | 0.08038719 |
| superpathway of 2, 3-butanediol biosynthesis | 0.82118883 | 0.07579728 |
| CDP-diacylglycerol biosynthesis I | 0.64735481 | **0.00286194** |
| CDP-diacylglycerol biosynthesis II | 0.64866571 | **0.00285964** |
| phosphatidylglycerol biosynthesis I (plastidic) | 0.19838205 | **0.00566778** |
| phosphatidylglycerol biosynthesis II (non-plastidic) | 0.19739406 | **0.0056508** |
| methylphosphonate degradation I | 0.26582967 | 0.22428167 |
| photorespiration | **0.03267111** | **0.02084123** |
| polyisoprenoid biosynthesis (E. coli) | 0.56986775 | **0.00302551** |
| glycogen biosynthesis I (from ADP-D-Glucose) | 0.87858682 | **0.00249676** |
| enterobacterial common antigen biosynthesis | 0.59964518 | 0.68531962 |
| starch biosynthesis | 0.57234664 | 0.53569788 |
| chondroitin sulfate degradation I (bacterial) | 0.22681643 | 0.7211897 |
| glycogen degradation I (bacterial) | 0.13926751 | 0.15750571 |
| glycogen degradation II (eukaryotic) | 0.43324409 | **0.02584287** |
| heparin degradation | 0.43918053 | 0.82759741 |
| mannan degradation | **0.00400038** | **0.00417768** |
| starch degradation III | 0.85048624 | 0.17320325 |
| starch degradation V | 0.2376282 | 0.12951 |
| superpathway of 5-aminoimidazole ribonucleotide biosynthesis | 0.38980652 | **0.00308138** |
| superpathway of adenosine nucleotides de novo biosynthesis I | 0.38709529 | **0.00298597** |
| superpathway of adenosine nucleotides de novo biosynthesis II | 0.38400489 | **0.00298579** |
| superpathway of guanosine nucleotides de novo biosynthesis I | 0.36492833 | **0.00297698** |
| superpathway of guanosine nucleotides de novo biosynthesis II | 0.36459532 | **0.00297631** |
| superpathway of purine nucleotides de novo biosynthesis I | 0.40082199 | **0.00299561** |
| superpathway of purine nucleotides de novo biosynthesis II | **0.0440428** | 0.63922021 |
| guanosine deoxyribonucleotides de novo biosynthesis II | 0.43035308 | **0.00296044** |
| guanosine ribonucleotides de novo biosynthesis | 0.33006759 | **0.00312631** |
| inosine-5'-phosphate biosynthesis I | 0.40031755 | **0.00301399** |
| inosine-5'-phosphate biosynthesis III | 0.71087113 | **0.00298145** |
| 5-aminoimidazole ribonucleotide biosynthesis I | 0.47016477 | **0.00298065** |
| 5-aminoimidazole ribonucleotide biosynthesis II | 0.38786112 | **0.00306827** |
| adenine and adenosine salvage III | 0.38344151 | **0.00417927** |
| adenosine deoxyribonucleotides de novo biosynthesis II | 0.43124928 | **0.002962** |
| adenosine ribonucleotides de novo biosynthesis | 0.35458465 | **0.00374178** |
| adenosine nucleotides degradation II | 0.89868366 | **0.00253934** |
| guanosine nucleotides degradation III | 0.87081214 | **0.00276689** |
| purine nucleotides degradation II (aerobic) | 0.34896243 | **0.00275482** |
| purine ribonucleosides degradation | 0.10288627 | 0.07478962 |
| urate biosynthesis/inosine 5'-phosphate degradation | 0.82355117 | **0.00254223** |
| pyrimidine deoxyribonucleosides salvage | 0.29633242 | **0.00380353** |
| pyrimidine deoxyribonucleotide phosphorylation | 0.29255821 | **0.00552417** |
| pyrimidine deoxyribonucleotides biosynthesis from CTP | 0.22871544 | 0.57863853 |
| pyrimidine deoxyribonucleotides de novo biosynthesis I | 0.689667 | **0.00508746** |
| pyrimidine deoxyribonucleotides de novo biosynthesis II | **0.04923612** | 0.64467539 |
| pyrimidine deoxyribonucleotides de novo biosynthesis III | **0.00529291** | 0.15936214 |
| pyrimidine deoxyribonucleotides de novo biosynthesis IV | 0.27064702 | 0.6115551 |
| superpathway of pyrimidine deoxyribonucleoside salvage | 0.72058608 | **0.00397449** |
| superpathway of pyrimidine deoxyribonucleotides de novo biosynthesis | 0.68733356 | **0.00375423** |
| superpathway of pyrimidine deoxyribonucleotides de novo biosynthesis (E. coli) | 0.61819359 | **0.0037948** |
| superpathway of pyrimidine nucleobases salvage | 0.48938733 | **0.00306841** |
| superpathway of pyrimidine ribonucleosides salvage | 0.89626772 | **0.00286424** |
| superpathway of pyrimidine ribonucleotides de novo biosynthesis | 0.42296762 | **0.00324726** |
| UMP biosynthesis | 0.47788375 | **0.00369806** |
| superpathway of pyrimidine deoxyribonucleosides degradation | 0.11182019 | **0.00393842** |
| isoprene biosynthesis II (engineered) | 0.09212197 | 0.09834128 |
| enterobactin biosynthesis | 0.84272899 | 0.09870744 |
| ergothioneine biosynthesis I (bacteria) | **0.00416832** | 0.10542723 |
| aerobactin biosynthesis | **0.00118893** | 0.91496833 |
| coumarins biosynthesis (engineered) | 0.29208355 | 0.34661836 |
| methylerythritol phosphate pathway I | 0.29598326 | **0.00334352** |
| methylerythritol phosphate pathway II | 0.29776151 | **0.0033207** |
| mevalonate pathway I | 0.13023801 | 0.09082244 |
| mevalonate pathway II (archaea) | 0.09169619 | 0.14514314 |
| preQ0 biosynthesis | 0.78036761 | **0.01884218** |
| spirilloxanthin and 2, 2'-diketo-spirilloxanthin biosynthesis | 0.76507186 | 0.70938832 |
| superpathway of geranylgeranyl diphosphate biosynthesis II (via MEP) | 0.22765265 | **0.00346611** |
| superpathway of geranylgeranyldiphosphate biosynthesis I (via mevalonate) | 0.13212285 | 0.08786386 |
| taxadiene biosynthesis (engineered) | 0.6111768 | **0.00639474** |
| gluconeogenesis I | 0.5724431 | **0.00296946** |
| O-antigen building blocks biosynthesis (E. coli) | 0.07406319 | **0.02160324** |
| sucrose biosynthesis I (from photosynthesis) | 0.94403546 | **0.00269963** |
| sucrose biosynthesis III | 0.91486941 | **0.0027492** |
| ADP-L-glycero-beta-D-manno-heptose biosynthesis | 0.54828441 | 0.71681569 |
| fucose degradation | **0.01090621** | **0.04217308** |
| galactose degradation I (Leloir pathway) | **0.03689592** | **0.00519899** |
| glucose and glucose-1-phosphate degradation | 0.48405081 | **0.00291593** |
| lactose and galactose degradation I | 0.11905492 | 0.41095196 |
| L-arabinose degradation IV | 0.09541856 | 0.05059484 |
| 1, 5-anhydrofructose degradation | **0.00801188** | 0.06958472 |
| glucose degradation (oxidative) | 0.45873404 | 0.84565424 |
| L-rhamnose degradation I | **0.01061752** | **0.04444065** |
| L-rhamnose degradation II | 0.67250141 | 0.58110086 |
| sucrose degradation II (sucrose synthase) | 0.44854032 | 0.21956713 |
| sucrose degradation III (sucrose invertase) | 0.48057489 | **0.0029983** |
| sucrose degradation IV (sucrose phosphorylase) | 0.47867368 | **0.00352182** |
| superpathway of glucose and xylose degradation | 0.62476673 | 0.74800178 |
| ketogluconate metabolism | 0.09011046 | 0.33417015 |
| 4-deoxy-L-threo-hex-4-enopyranuronate degradation | **0.02334362** | **0.00289457** |
| D-fructuronate degradation | 0.0820414 | **0.00278451** |
| D-galactarate degradation I | **0.01124341** | 0.25614435 |
| D-galacturonate degradation I | 0.06318201 | **0.00282076** |
| D-glucarate degradation I | **0.01063266** | 0.33161505 |
| myo- chiro- and scillo-inositol degradation | **0.0074449** | 0.15861877 |
| sulfoglycolysis | 0.33614021 | 0.62257674 |
| superpathway of hexitol degradation (bacteria) | **0.01912119** | **0.01916557** |
| superpathway of beta-D-glucuronide and D-glucuronate degradation | 0.93317053 | **0.00250622** |
| anhydromuropeptides recycling | 0.82571186 | **0.01206404** |
| myo-inositol degradation I | **0.01859957** | 0.26845962 |
| dTDP-L-rhamnose biosynthesis I | 0.80303644 | **0.00297542** |
| CMP-3-deoxy-D-manno-octulosonate biosynthesis I | 0.19263185 | **0.00286204** |
| CMP-legionaminate biosynthesis I | **0.00402949** | **0.00425091** |
| dTDP-N-acetylthomosamine biosynthesis | 0.06388047 | 0.20659792 |
| GDP-D-glycero-?-D-manno-heptose biosynthesis | 0.91713293 | 0.79401793 |
| GDP-mannose biosynthesis | 0.28633882 | **0.00354871** |
| superpathway of GDP-mannose-derived O-antigen building blocks biosynthesis | 0.36225145 | **0.00612088** |
| superpathway of UDP-glucose-derived O-antigen building blocks biosynthesis | 0.88497595 | **0.00270247** |
| superpathway of UDP-N-acetylglucosamine-derived O-antigen building blocks biosynthesis | 0.47684861 | 0.4941859 |
| UDP-2,3-diacetamido-2,3-dideoxy-?-D-mannuronate biosynthesis | 0.12171922 | **0.04253264** |
| UDP-N-acetyl-D-glucosamine biosynthesis I | **0.03425827** | **0.03706558** |
| sulfate reduction I (assimilatory) | 0.64476631 | **0.00755299** |
| superpathway of sulfate assimilation and cysteine biosynthesis | 0.84045092 | **0.00296536** |
| superpathway of sulfolactate degradation | **0.0308435** | **0.0474453** |
| superpathway of sulfur oxidation (Acidianus ambivalens) | 0.42359113 | 0.16472642 |
| superpathway of taurine degradation | 0.64982235 | 0.66198641 |
| aspartate superpathway | 0.34549799 | **0.0035502** |
| superpathway of chorismate metabolism | 0.33738351 | 0.24193612 |
| superpathway of D-glucarate and D-galactarate degradation | **0.01144028** | 0.25525005 |
| superpathway of fucose and rhamnose degradation | 0.058241 | 0.33050802 |
| superpathway of glyoxylate bypass and TCA | 0.86562969 | **0.00424735** |
| superpathway of hexuronide and hexuronate degradation | 0.31001849 | **0.00245579** |
| superpathway of histidine, purine, and pyrimidine biosynthesis | 0.05419557 | 0.83059386 |
| superpathway of S-adenosyl-L-methionine biosynthesis | 0.27417678 | **0.00920492** |
| tetrapyrrole biosynthesis I (from glutamate) | 0.30827497 | **0.00298232** |
| tetrapyrrole biosynthesis II (from glycine) | 0.35874376 | **0.0063399** |
| arginine, ornithine and proline interconversion | 0.20897709 | 0.61407201 |
|  |  |  |
| Total number of statistically significant pathways | 104 | 223 |

| **Table S8** P-Values from P-Values from the Kruskal-Wallis (KW) and the Mann-Whitney (MW) tests on the 5 functional Metacyc bacterial pathways selected of those identified with Picrust2 across different sample types. Statistically significant differences (P-values < 0.05) are highlighted in bold. | | | | |
| --- | --- | --- | --- | --- |
| Metacyc pathway | Bare soil/  *Prasiola*-covered soil | | *Prasiola* soil/ *Prasiola* canopy |  |
|  | MW | MW | | KW |
| Methanogenesis | 0.190 | 0.086 | | **0.01645** |
| Autotrophic CO2 Fixation | 0.111 | **0.024** | | **0.005917** |
| Nitrogen Compound Metabolism | 0.33 | 0.73 | | 0.4449 |
| Photosynthesis | 0.29 | 0.29 | | 0.1429 |
| Sulfur Compound Metabolism | 0.19 | 0.90 | | 0.3499 |

**Table S9** P-Values obtained from Mann-Whitney test on identified fungal functional categories across both type of soils.

| Functional category | P-Values |
| --- | --- |
| Lichen parasite | 1 |
| Wood saprotroph | 1 |
| Litter saprotroph | 0.5556 |
| Animal parasite | 0.1905 |
| Dung saprotroph | 0.3061 |
| Mycoparasite | 0.2857 |
| Lichenised | 0.2663 |
| Unspecified saprotroph | 0.9048 |
| Plant pathogen | 0.5556 |
| Soil saprotroph | 0.5556 |
| Unclassified | 0.5556 |
